# Compartment-specific cAMP dysfunction in diabetic cardiomyopathy in hiPSC-derived cardiomyocytes: therapeutic potential of fibroblast growth factor 1

**DOI:** 10.1186/s12933-026-03288-9

**Published:** 2026-07-30

**Authors:** Kun Liu, Kazuki Osuka, Mengying Xu, Rhea Takhar, Neda Mohammadi, Adam Trevett, Jinyan Zhong, Ching-Wen Chang, Nan Wang, Xinyu Hu, Ruirui Dong, Shunan Zhang, YoonYoung Choi, Thamali Ayagama, Mingyu Li, Linna Zhou, Che-Mai Chang, Wan-Chen Huang, Wei-Chiao Chang, Neil Herring, Annika Winbo, Guoliang Hao, Jie Zhang, Dan Li

**Affiliations:** 1 Burdon Sanderson Cardiac Science Centre and BHF Centre of Research Excellence, Department of Physiology, Anatomy and Genetics, University of Oxford, Oxford, UK; 2 Institute of Electrophysiology, Henan Academy of Innovations in Medical Science, Zhengzhou, 450000 China; 3 Ningbo No.2 Hospital, Wenzhou Medical University, No.41, Xibei Street, Ningbo, Zhejiang China; 4 Master Program in Clinical Genomics and Proteomics, School of Pharmacy, Taipei Medical University, Taipei, Taiwan; 5 Institute of Medicinal Plant Development, Chinese Academy of Medical Sciences & Peking Union Medical College, Beijing, 100193 China; 6 Ellison Institute of Technology, Winchester House, Heatley Road, The Oxford Science Park, Littlemore, Oxford UK; 7 Single-Molecule Biology Core Lab, Institute of Cellular and Organismic Biology (ICOB), Academia Sinica, 128 Academia Road, Section 2, Nankang, Taipei, 115 Taiwan; 8 Department of Public Health and Clinical Medicine, Faculty of Medicine, Umeå University, Umeå, Sweden; 9 CAMS Oxford Institute, Peking Union Medical College, Chinese Academy of Medical Sciences, University of Oxford, Oxford, UK

**Keywords:** Diabetic cardiomyopathy, cAMP signaling, hiPSC-derived cardiomyocytes, PDE4DIP, Compartmentalization, FGF1, 3D cardiac microtissues

## Abstract

**Background:**

Diabetic cardiomyopathy (DCM) represents a distinct form of heart failure occurring independently of coronary artery disease, yet effective treatments remain limited. While cAMP signaling alterations have been reported in diabetic hearts, the compartment-specific consequences across distinct subcellular pools remain poorly understood. We investigated cytosolic and mitochondrial cAMP microdomain dysfunction in DCM, focusing on ADCY2 and PDE4DIP as potential regulators, and evaluated fibroblast growth factor 1 (FGF1) as a therapeutic intervention using integrated two-dimensional (2D) and three-dimensional (3D) human induced pluripotent stem cell (hiPSC)-derived cardiac models.

**Methods:**

Healthy hiPSC-derived cardiomyocytes were exposed to a diabetic-like environment (high glucose, endothelin-1 and cortisol) in 2D and 3D culture. Two-dimensional studies included bulk RNA sequencing, FRET-based cAMP measurements in cytosolic and mitochondrial outer membrane compartments, and microelectrode array electrophysiology. Three-dimensional cardiac microtissues were assessed by optical mapping to characterize spatial calcium dynamics and conduction properties.

**Results:**

DCM exhibited compartment-specific cAMP dysregulation associated with *ADCY2* downregulation and *PDE4DIP* upregulation. Cytosolic compartments showed preserved baseline cAMP but attenuated forskolin responsiveness, while the mitochondrial outer membrane displayed both reduced baseline cAMP and impaired stimulated responses. Transcriptomic analysis revealed transcriptional signatures consistent with metabolic remodeling, including upregulated fatty acid oxidation machinery, Complex I dysfunction, and elevated phospholamban and *CASQ2* expression. 3D optical mapping demonstrated reduced calcium transient amplitude, prolonged transient duration, and severely impaired calcium propagation velocity. FGF1 treatment normalized PDE4DIP expression and restored cAMP dynamics in 2D cultures and partially improved calcium transient amplitude in 3D microtissues, though conduction defects persisted.

**Conclusions:**

Compartment-specific impairment of cAMP microdomains is associated with DCM pathogenesis. Integrated 2D–3D assessment reveals that FGF1 improves cAMP signaling but does not fully reverse metabolic, electrophysiological, or structural abnormalities, highlighting the need for combination therapies targeting both cAMP pathway integrity and conduction restoration.

**Graphical abstract:**

**Figure:**
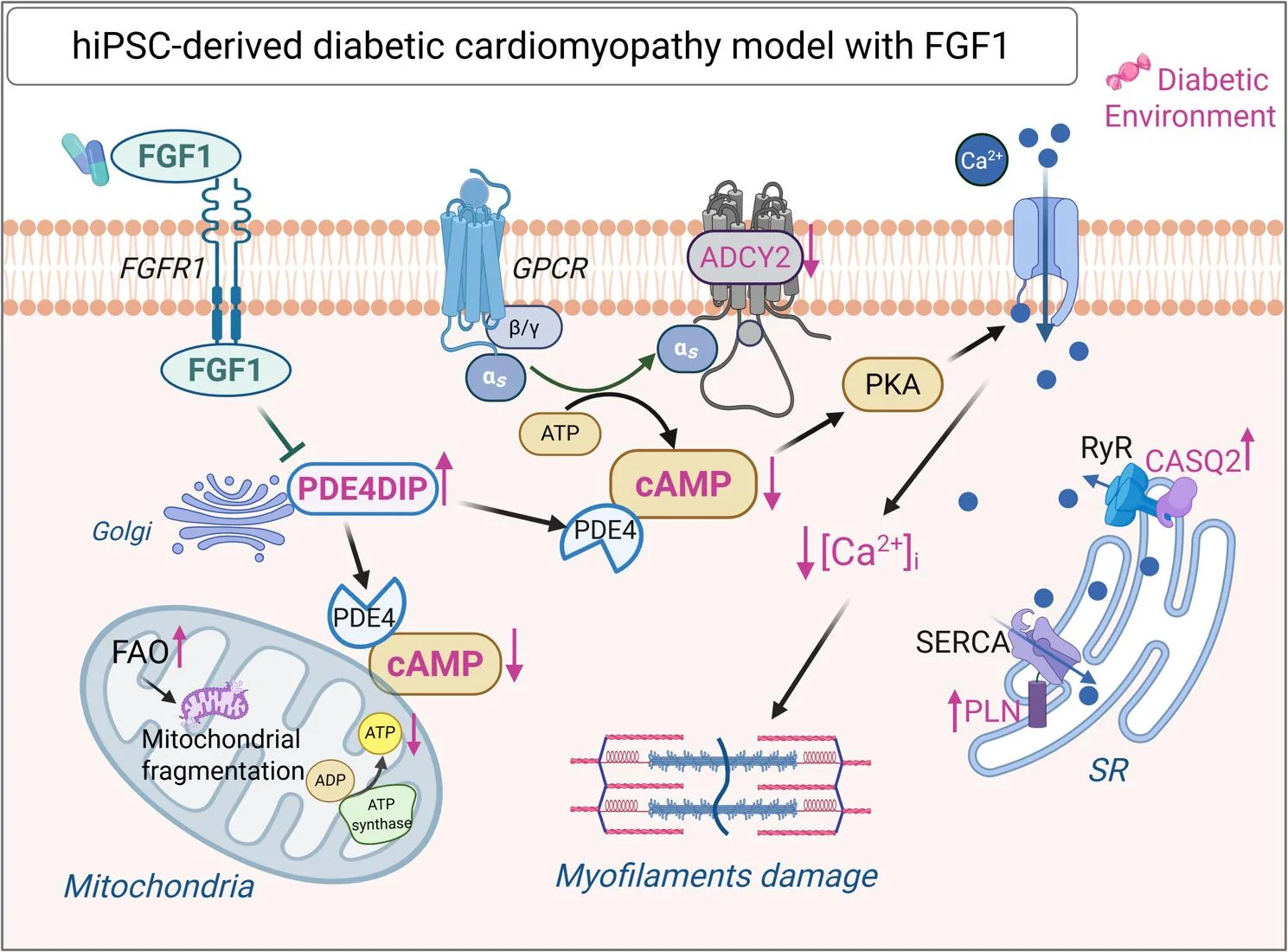

**Supplementary Information:**

The online version contains supplementary material available at https://doi.org/10.1186/s12933-026-03288-9.

## Research insights

**What is currently known about this topic?**

- Diabetic cardiomyopathy causes contractile dysfunction, involves cAMP dysregulation, and current models, including 2D cultures, capture limited aspects of disease complexity.

**What is the key research question?**

- Does diabetes cause compartment-specific cAMP dysfunction, and can FGF1 restore this signaling?

**What is new?**

- Diabetic cardiomyopathy shows compartment-specific cAMP dysregulation across subcellular pools.
- PDE4DIP is identified as a potential therapeutic target associated with metabolic stress to cAMP dysfunction.
- 3D microtissues reveal persistent conduction defects despite cAMP restoration, showing mixed reversibility.

**How might this study influence clinical practice?**

- Early cAMP pathway targeting may prevent progression from reversible to fixed diabetic cardiomyopathy defects.

## Background

Diabetes mellitus is a global health crisis with widespread cardiovascular implications, affecting 9.3% of the global population, with projections indicating a 25% increase by 2030 [1]. Among its cardiovascular complications, diabetic cardiomyopathy (DCM) represents a distinct pathophysiological entity occurring independently of coronary artery disease or hypertension, characterized by early diastolic dysfunction, progressive ventricular remodeling, and eventual heart failure [2, 3]. Despite extensive clinical characterization, effective DCM prevention and treatment remain elusive.

The transition from diabetes to DCM involves complex metabolic reprogramming wherein cardiomyocytes develop pathological reliance on fatty acid oxidation, leading to mitochondrial dysfunction, lipotoxicity, and impaired insulin signaling [4, 5]. Central to this pathophysiological cascade is the cyclic adenosine monophosphate (cAMP) signaling network, which orchestrates cardiac excitation-contraction coupling through spatially organized microdomains defined by adenylyl cyclases and phosphodiesterases [6, 7]. Recent evidence demonstrates that cAMP operates through highly compartmentalized nanodomains that precisely regulate calcium handling, mitochondrial energetics, and β-adrenergic responsiveness [8–10].

Alterations in cardiac cAMP signalling have been reported in diabetic hearts, including time-dependent changes in Epac1/2 expression and altered calcium-handling protein phosphorylation [11]. However, whether these changes reflect the compartment-specific consequences across distinct subcellular pools, particularly cytosolic versus mitochondrial cAMP compartments remain poorly understood. Human induced pluripotent stem cell (hiPSC) technology now enables generation of cardiomyocytes that can be cultured under diabetic conditions to model DCM pathophysiology, facilitating systematic investigation of key pathophysiological features while enabling genetic and pharmacological manipulation [12, 13].

Fibroblast growth factor 1 (FGF1) has emerged as a compelling therapeutic candidate given its reduced expression in type 2 diabetes patients. Its levels are positively correlated with cardiac function, and FGF1 has been implicated in maintaining mitochondrial homeostasis, reducing oxidative stress, and modulating metabolic pathways [14, 15]. However, whether FGF1’s therapeutic mechanisms extend to restoration of compartment-specific cAMP signaling dysfunction in DCM, and whether such intervention can reverse the electrophysiological and calcium handling abnormalities characteristic of DCM, remains unexplored.

**Fig. 1:**
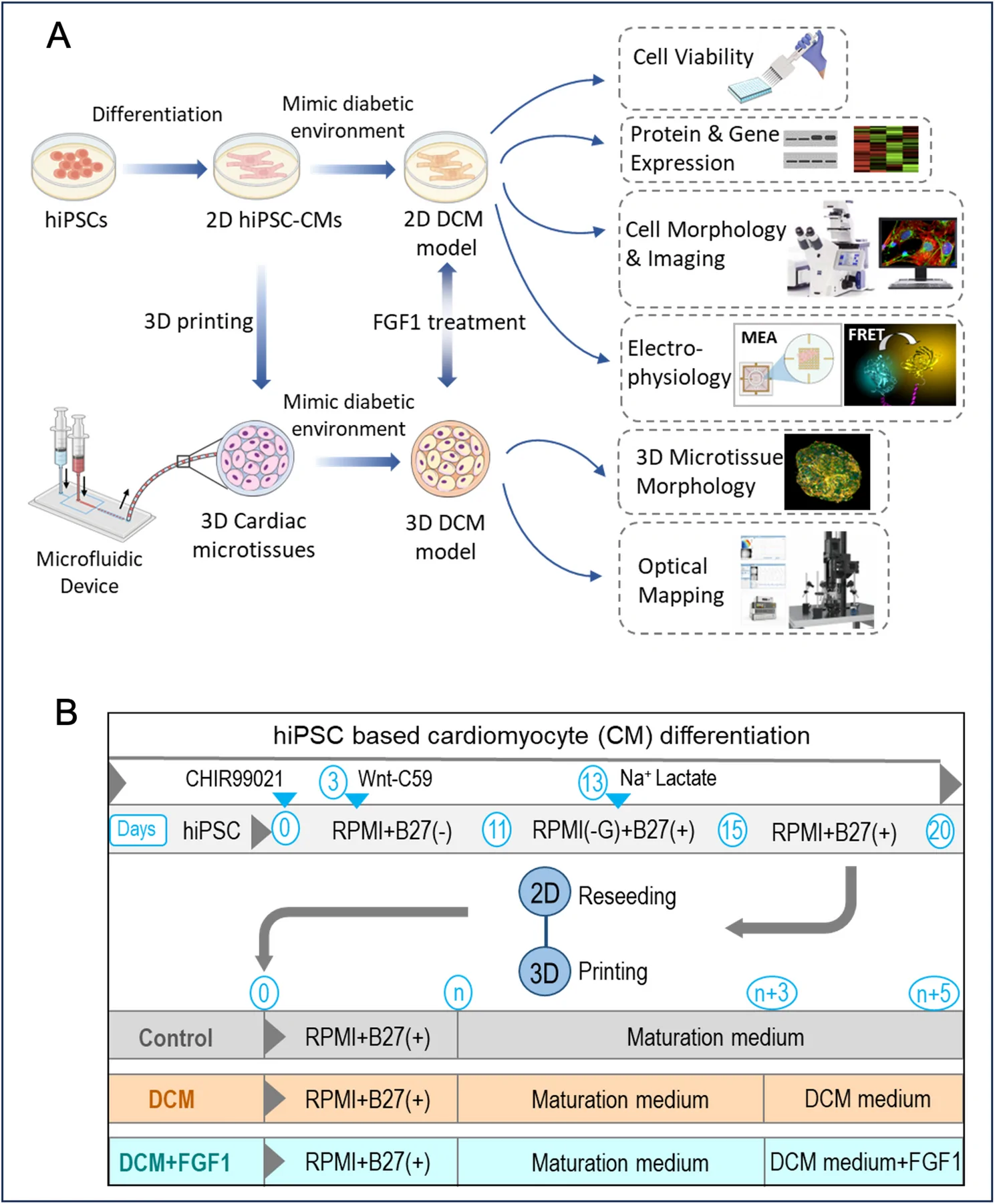
Experimental workflow and treatment scheme. **A** Overview of hiPSC-cardiomyocyte differentiation and generation of 2D monolayers and 3D cardiac tissues, followed by downstream assays. **B** Schematic summary of the differentiation timeline and treatment allocation. After differentiation into cardiomyocytes, cells were reseeded as monolayers or printed into 3D cardiac tissues between days 15 and 20, maintained in RB+ medium for > 10 days, and then transferred to maturation medium for 3 days. Thereafter, cells and microtissues were assigned to Control (standard maturation medium), DCM (maturation medium supplemented with 11 mM glucose, 10 nM endothelin-1, and 1 µM cortisol for 2 days), or DCM + FGF1 (DCM medium supplemented with 1 ng/mL FGF1 for 2 days) groups. Detailed experimental procedures are provided in the Methods section

Here, we developed hiPSC-derived cardiomyocyte (hiPSC-CM) models exposed to a diabetic-like environment and employed integrated approaches combining bulk RNA-sequencing, FRET-based compartment-specific cAMP measurements in 2D cultures, MEA electrophysiology, and 3D optical mapping to characterize cAMP microdomain dysfunction in DCM (Fig. 1A). We evaluated FGF1 as a therapeutic intervention and assessed its effects on cAMP signaling restoration and functional rescue across multiple experimental platforms.

## Materials and methods

### Human-iPSCs culture and maintenance

Two healthy hiPSC lines (Gibco™ and WT control) were used for cardiac myocyte differentiation in this study. Gibco™ episomal hiPSC line from Thermo Fisher Scientific had ethical licence/patient consent: − 45 CFR Part 46, 42 CFR Part 482. WT control hiPSC line was approved by Umeå, Sweden, and Health and Disability Ethics Committees: 17/NTA/226 (2024). All experiments were performed in accordance with UK guidelines and regulations and as set out in the REC. hiPSCs were thawed and expanded on Matrigel-coated plates (Corning, UK) in mTeSR1 medium (STEMCELL Technologies, UK) until reaching 90–95% confluence prior to differentiation. All experiments were performed using the Gibco™ episomal hiPSC line, except for Supplementary Fig. S4, which shows optical mapping data from the wild-type line.

### Differentiation of hiPSC-derived cardiomyocytes (hiPSC-CMs)

Cardiomyocyte differentiation was induced using a small molecule-based protocol (Fig. 1B). On day 0, medium was switched to RB- medium [RPMI-1640 (Gibco, Thermo Fisher Scientific, Altrincham, Cheshire, UK) supplemented with B-27 minus insulin (Gibco, Thermo Fisher Scientific, Altrincham, Cheshire, UK)] containing 6 µM CHIR99021 (Tocris Bioscience, UK) for 48 h. On day 2, medium was replaced with fresh RB- medium. On day 3, cells were treated with RB- containing 2.5 µM Wnt-C59 (Tocris Bioscience, UK) for 48 h. From day 5 onwards, cells were maintained in RB- with medium changes every two days. Metabolic purification was performed from days 11 to 14 using glucose-free RPMI (Gibco, UK) supplemented with B-27 supplement minus insulin and 5 mM sodium L-lactate (Sigma, Darmstadt, Germany). Subsequently, hiPSC-CMs were maintained in RB+ medium [RPMI-1640 with B-27 Supplement (Gibco, UK)]. For experiments, cells were dissociated using TrypLE Select Enzyme (Gibco, UK) and replated onto Matrigel-coated surfaces. hiPSC-derived cardiomyocytes from the Gibco line were used at 30–35 days post-differentiation, and wild-type line cardiomyocytes were used at 44 days post-differentiation at the time of experimentation.

### hiPSC-derived diabetic cardiomyopathy model

The DCM model was adopted from Drawnel et al. [12]. Following 15 days of differentiation, hiPSC-CMs were dissociated and replated, and subsequently divided into control and DCM model groups. To promote metabolic maturation, hiPSC-CMs were cultured in a defined cardiomyocyte maturation medium consisting of DMEM (Thermo Fisher, UK) supplemented with 10 mM HEPES (Thermo Fisher, UK), 2 mM L-carnitine (Sigma, UK), 5 mM creatine (Sigma, UK), 5 mM taurine (Sigma, UK), 1× MEM Non-Essential Amino Acids (Thermo Fisher, UK), 1× Insulin-Transferrin-Selenium (ITS-G) (Thermo Fisher, UK), 1× linoleic acid–oleic acid–albumin (Sigma, UK), and 3 mM D-glucose (Sigma, UK). To establish a diabetic phenotype (DCM model), hiPSC-CMs were first cultured in maturation medium for 3 days, then followed by 2 days to a diabetic medium consisting of maturation medium supplemented with 11 mM D-glucose, 10 nM endothelin-1 (Sigma, USA), and 1 µM cortisol (Tocris Bioscience, UK). For the treatment group, 1 ng/mL recombinant human FGF1 (Miltenyi Biotec, UK) was added to the diabetic medium for the final 2 days of culture prior to experiments.

### 3D microtissue fabrication via droplet printing

hiPSC-CMs were dissociated, pelleted, and resuspended in ice-cold growth factor reduced Matrigel (40 million cells/mL, Corning, Loughborough, UK) to form bioink. Microtissues were fabricated using a custom microfluidic droplet generator as previously described [16, 17]. Cell-laden Matrigel droplets were generated, incubated at 37 °C for 2 h for gelation, and then transferred to 48-well ultra-low attachment plates containing cardiomyocyte culture medium plus 10 µM Y-27,632 Rock inhibitor (Cell Guidance Systems, Cambridge, UK) and 100 U/ml penicillin–streptomycin (Thermo Fisher, UK), maintained at 37 °C, 5% CO_2_ for the first 24 h. The next day, the medium was exchanged with cardiomyocyte culture medium without additives. A fresh medium change was then performed every 3 days.

### Immunofluorescence staining and morphometric analysis

hiPSC-CMs were seeded onto glass-bottom FluoroDishes (World Precision Instruments, USA) at appropriate densities. After 2–5 days, cells were fixed with 4% formaldehyde, permeabilized with 0.1% Triton X-100 (Thermo Fisher, UK), blocked with 6% donkey serum (Sigma, UK), and stained with primary antibodies against cardiac troponin T (cTnT, Ab-cam, UK, 1:200), α-actinin (Sigma, UK, 1:800) and TOM20 (Thermo Fisher, UK, 1:200). Nuclei were counterstained with Hoechst 33,342 (Thermo Fisher, UK, 1:10,000). The samples were then incubated with appropriate secondary antibodies (Alexa Fluor 488, invitrogen (anti-mouse or -rabbit), 1:1000; Alexa Fluor 594, invitrogen (anti-mouse or -rabbit), 1:800, UK). Confocal fluorescence images were acquired using a Zeiss LSM 880 Airy Scan microscope (Germany). Cell and nuclear areas were quantified using ImageJ software. Individual cells were manually outlined, while nuclear regions were segmented automatically based on intensity thresholding.

### Bulk RNA-sequencing

Bulk RNA-sequencing was performed using total RNA extracted with the RNeasy Kit (QIA-GEN) and sequenced on the BGISEQ-500 platform. Four independent pooled RNA samples per group were prepared from a single differentiation batch, and each sample was collected from cells pooled from two 12-well plates. Raw gene count tables were downloaded from the BGI portal, converted to CSV, and processed by standardising gene identifiers and removing genes with zero counts across all samples. Sample condition labels (control or DCM) were inferred from file names. The filtered integer count matrix was used for downstream analysis in DESeq2, where variance-stabilising transformation (VST) was applied and principal component analysis (PCA) performed to assess sample clustering and detect outliers. Differential expression between DCM and control groups was computed using DESeq2 with shrinkage-adjusted log2 fold changes (apeglm), and genes meeting FDR < 0.05 and absolute log2FC > 1 were classified as differentially expressed for upstream analyses, while a more permissive threshold of FDR < 0.05 and absolute log2FC > 0.5 was applied for downstream functional and pathway analyses. Functional enrichment of up- and down-regulated genes was carried out using clusterProfiler for Gene Ontology and KEGG pathways. Additionally, Gene set enrichment analysis (GSEA) was performed on ranked gene lists using MSigDB gene sets, and leading-edge genes from significantly enriched pathways were used for downstream visualisation and analysis.

### Cell viability assay

Cellular ATP levels, correlating with viability, were measured using the CellTiter-Glo 2.0 Assay (Promega, USA). hiPSC-CMs were seeded in 96-well plates at a density of 4 × 10^4^ cells per well and cultured in either control or diabetic medium. Luminescence was quantified by BioTek Cytation 5 (USA) according to the manufacturer’s instructions.

### Glucose reuptake assay

Glucose uptake capacity was assessed using the Glucose Uptake-Glo Assay (Promega, J1342). hiPSC-CMs were plated in 96-well plates (4 × 10^4^ cells/well). 2-Deoxyglucose (2-DG) uptake was measured by recording luminescence (relative light units, RLU) (BioTek Cytation 5 (USA) as per the kit instructions.

### Mitochondrial membrane potential measurement

Mitochondrial membrane potential (∆Ψm) was assessed using the JC-1 dye (Thermo Fisher, USA). hiPSC-CMs were incubated with 2 µM JC-1 for 30 min at 37 °C. Fluorescence images (red aggregates and green monomers) were acquired on a Zeiss LSM 880 confocal microscope (Germany). The red/green fluorescence intensity ratio was calculated using ImageJ.

### RNA extraction and quantitative RT-PCR

Total RNA was extracted using the RNeasy Mini Kit (QIAGEN, UK). cDNA was synthesized from 1 µg RNA using the iScript cDNA Synthesis Kit (Bio-Rad, UK). Quantitative PCR was performed in 20 µL reactions containing TaqMan Universal PCR Master Mix (Applied Biosystems, UK), cDNA, and TaqMan Gene Expression Assays for *NPPB* (Hs00173590_m1), *NPPA* (Hs00383230_g1), *MYH7* (Hs01110632_m1), *PDE4DIP* (Hs00206200_m1), *ADCY2* (Hs01058848_m1) and GAPDH (Hs02786624_g1) (Thermo Fisher, UK). Thermocycling was conducted on an ABI Prism 7000 system (Applied Biosystems, USA). Relative gene expression was calculated using the comparative ∆∆Ct method with *GAPDH* as the endogenous control.

### Western blotting

Cells were lysed in RIPA buffer (Merck, Darmstadt, Germany) containing protease and phosphatase inhibitors. Proteins (30 µg) were separated on 4–12% Bis-Tris gels (Bio-Rad, USA) and transferred to PVDF membranes (Bio-Rad, USA). Membranes were blocked and incubated overnight at 4 °C with primary antibodies against PDE4DIP (Abcam, UK, 1:1000) and GAPDH (Proteintech, UK; loading control, 1:5000). Following incubation with HRP-conjugated secondary antibodies (Goat anti-Mouse IgG (H + L), 1:5000; Goat anti-Rabbit IgG (H + L), 1:5000; Thermo Fisher, UK), bands were visualized using Amersham ECL Select Western Blotting Detection Reagent (Amersham, USA) and quantified with ImageJ.

### Real-time cAMP dynamics using FRET imaging

hiPSC-CMs were prepared as a monolayer and transduced with an adenoviral vector encoding the cytosolic cAMP biosensor Epac-S^H187^, or targeted to the outer mitochondrial membrane (OMM) cAMP biosensor OMM-H187 by overnight incubation at 37 °C, 5% CO_2_ before FRET measurements [8]. Viral volume was adjusted by cell amount. After overnight expression, cells were bathed in Tyrode’s solution (135 mM NaCl, 4.5 mM KCl, 20 mM HEPES, 11 mM glucose, 1 mM MgCl_2_, 2 mM CaCl_2_, pH 7.4) to establish a baseline. cAMP production was stimulated by sequential application of 10 nM and 25 µM forskolin (Cayman Chemical, USA), with 100 µM IBMX (Sigma, Darmstadt, Germany) added for saturation. FRET ratio images (480 nm/535 nm emission upon 430 nm excitation) were acquired every 15 s using a Nikon Eclipse Ti microscope (Japan). The mean FRET response was plotted against time and expressed as the percentage of ΔR/R0, where ΔR = R – R0 and R0 is the mean of the four FRET ratios preceding the addition of the first drug, and R is the ratio at time = t seconds.

### Micro-electrode array (MEA) recording

MEA measurements were performed as previously described [17]. Briefly, hiPSC-CMs were reseeded onto Matrigel-coated MEA plates (MappingLab, UK). Extracellular field potentials were recorded on days 3–5 post-seeding using MEA hardware (MappingLab, UK). Recordings were obtained at baseline (0, 5, 10 min) and after acute administration of 100 nM isoproterenol (ISO, Sigma, USA)) at 1 and 5 min. Data were analyzed using EMapScope5 software (MappingLab, UK). Channels with optimal signal-to-noise ratios were selected for conduction velocity mapping.

### Optical mapping of 3D microtissues

3D hiPSC-CMs constructs were loaded with 10 µM Rhod-2 AM (Abcam, UK) for 20 min. Calcium transients were recorded at baseline (0, 5, 10 min) and after acute administration of 100 nM isoproterenol (ISO) at 1 and 5 min. using an optical mapping system (OMS-PSCIE-2002, MappingLab, UK). Parameters including activation time, calcium propagation velocity, amplitude, rise time, and calcium transient duration (CTD) were analyzed with OMapScope software (MappingLab, UK).

### Statistical analysis

Data are presented as mean ± standard error of the mean (SEM). Statistical analyses were performed using GraphPad Prism 9.0. Comparisons between two groups used Student’s t-test or the Mann–Whitney U test. Multiple group comparisons used one-way or two-way ANOVA with appropriate post-hoc tests. Significance levels are denoted as *p < 0.05, **p < 0.01, ***p < 0.001, ****p < 0.0001, and ns: not significant.

## Results

### Bulk RNA sequencing identifies canonical diabetic cardiomyopathy markers and pathway remodeling

**Fig. 2:**
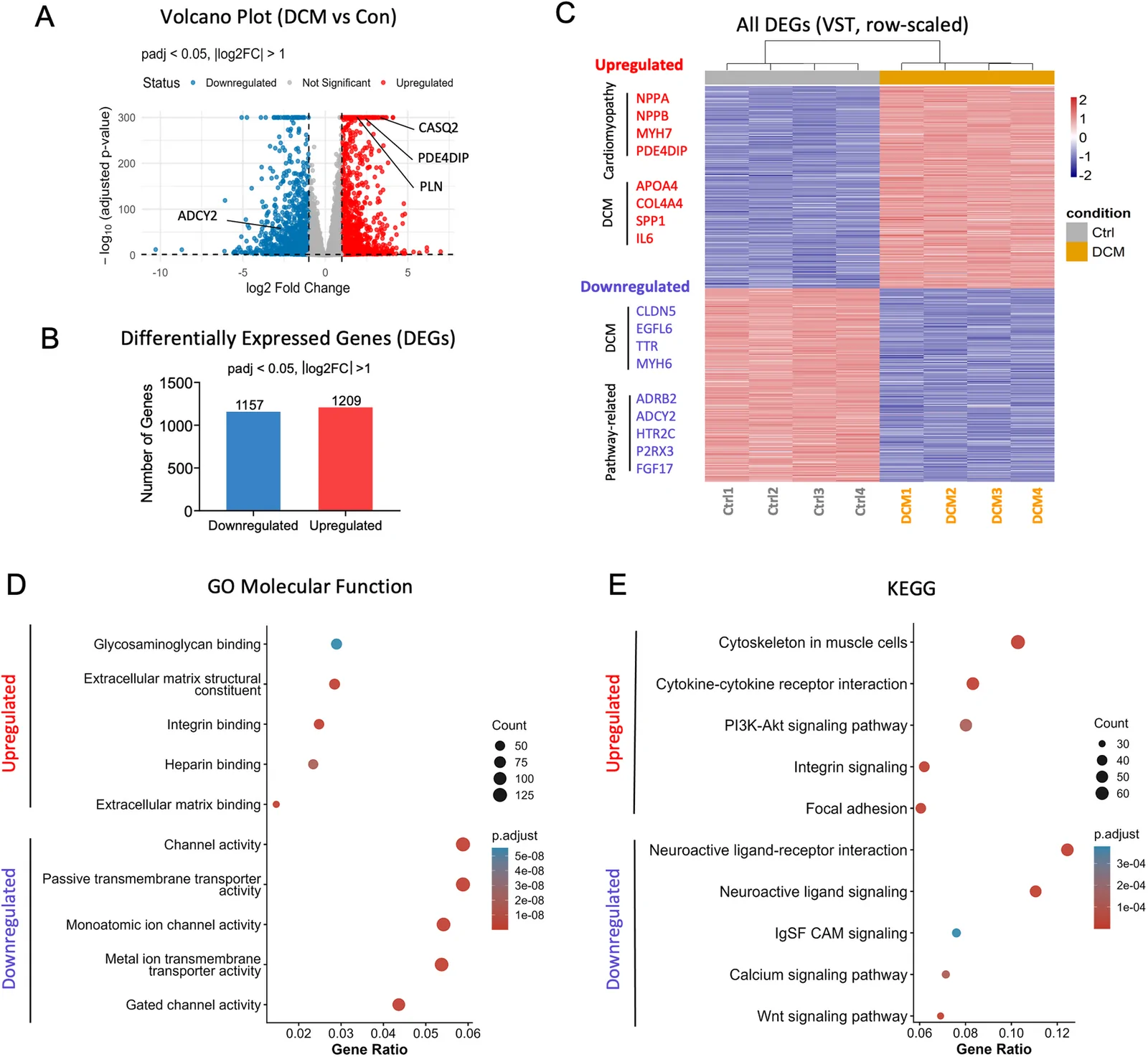
Bulk RNA sequencing results. **A** Volcano plot showing the relationship between log₂ fold change and statistical significance (− log_10_ adjusted *p*-value) for all expressed genes, with significantly upregulated and downregulated genes highlighted. **B** Bar plot summarising the number of upregulated and downregulated differentially expressed genes, defined using an adjusted *p*-value < 0.05 and an absolute log₂ fold change > 1. **C** Heatmap of all differentially expressed genes ranked by adjusted *p*-value demonstrates diabetic cardiomyopathy-related, cardiomyopathy-related, and pathway-related genes. **D** Gene ontology (GO) over-representation analysis for top 5 upregulated and downregulated enriched molecular function terms. Terms are ranked by statistical significance, with dot size indicating gene ratio and colour representing adjusted *p*-value. **E** KEGG pathway over-representation analysis for top 5 upregulated and downregulated pathways displayed according to gene ratio and statistical significance

RNA-sequencing analysis revealed extensive transcriptional remodeling in DCM cardiomyocytes compared to control conditions. Volcano plot analysis identified 2366 significantly differentially expressed genes (adjusted *p*-value < 0.05, Fig. 2A), with 1209 genes upregulated and 1157 genes downregulated in DCM (Fig. 2B). Among the upregulated genes were key markers of cardiac stress and remodeling including *PDE4DIP*, phospholamban (*PLN*), and calsequestrin 2 (*CASQ2*), while downregulated genes included components of the cAMP synthesis pathway such as *ADCY2* (Fig. 2A).

Expression heatmap of differentially expressed genes (*n* = 2366) shows robust transcriptional reprogramming in DCM cardiomyocytes compared to control, with distinct clustering of co-regulated gene modules (Fig. 2C). Among the most strongly upregulated cardiomyocyte-enriched genes, we detected *NPPA*, *NPPB*, and *MYH7* which constitute a well-established signature for diseased cardiomyocytes. *NPPA* and *NPPB*, which encode atrial and B type natriuretic peptides respectively, are classic cardiac stress genes that are reactivated and upregulated in hypertrophy and heart failure, while *MYH7*, encoding the B-myosin heavy chain, is a key sarcomeric gene, and its increased expression are closely associated with hypertrophic remodeling. The *APOA4* (apolipoprotein A-IV), an intramyocardial chylomicron-associated apolipoprotein, showed significant increase, consistent with dysregulated lipid handling in diabetic cardiomyopathy [18–20]. Upregulation of *COL4A4* reflects enhanced extracellular matrix deposition and basement membrane remodeling [18, 21], while *SPP1* (osteopontin) exhibited marked overexpression in line with its established contribution to cardiac fibrosis, hypertrophy, and inflammatory remodeling in diabetes related cardiac disease [22–24].

Conversely, several strongly downregulated genes were associated with endothelial function (*CLDN5*), angiogenesis (*EGFL6*), and cardiometabolic signaling (*TTR*). *CLDN5*, a canonical endothelial tight junction marker, was significantly reduced, suggesting impaired endothelial-associated signaling within the diabetic myocardium [25]. *EGFL6*, an important pro-angiogenic factor, was also downregulated in a pattern consistent with defective reparative angiogenesis [26, 27]. Additionally, *TTR* showed significant reduction and has previously been linked to cardiometabolic dysfunction in type 2 diabetes [28]. These gene-level changes collectively recapitulate molecular features characteristic of heart failure and diabetic cardiomyopathy.

Gene Ontology molecular function analysis revealed distinct functional categories enriched among upregulated and downregulated gene sets in DCM (Fig. 2D). Upregulated genes were significantly enriched for glycosaminoglycan binding, extracellular matrix structural constituent, integrin binding, heparin binding, and extracellular matrix binding, indicating enhanced extracellular matrix remodeling and growth factor signaling responses. In contrast, downregulated genes showed strong enrichment for channel activity, passive transmembrane transporter activity, monoatomic ion channel activity, metal ion transmembrane transporter activity, and gated channel activity, suggesting impaired ionic transport and compromised excitation-contraction coupling in DCM cardiomyocytes.

KEGG pathway analysis further elucidated the biological processes underlying DCM pathogenesis (Fig. 2E). Upregulated genes were most significantly enriched in cytoskeleton in muscle cells, along with cytokine-cytokine receptor interaction, PI3K-Akt signaling pathway, integrin signaling, and focal adhesion pathways, consistent with structural remodeling and inflammatory responses. Downregulated pathways included neuroactive ligand-receptor interaction, neuroactive ligand signaling, IgSF CAM signaling, calcium signaling pathway and Wnt signaling pathway. Together, these data indicate coordinated suppression of neuro-signaling programmes and activation of structural, integrin-mediated, and inflammatory remodeling pathways.

### Transcriptomic analysis reveals coordinated suppression of calcium- and cAMP-linked signaling programmes

**Fig. 3:**
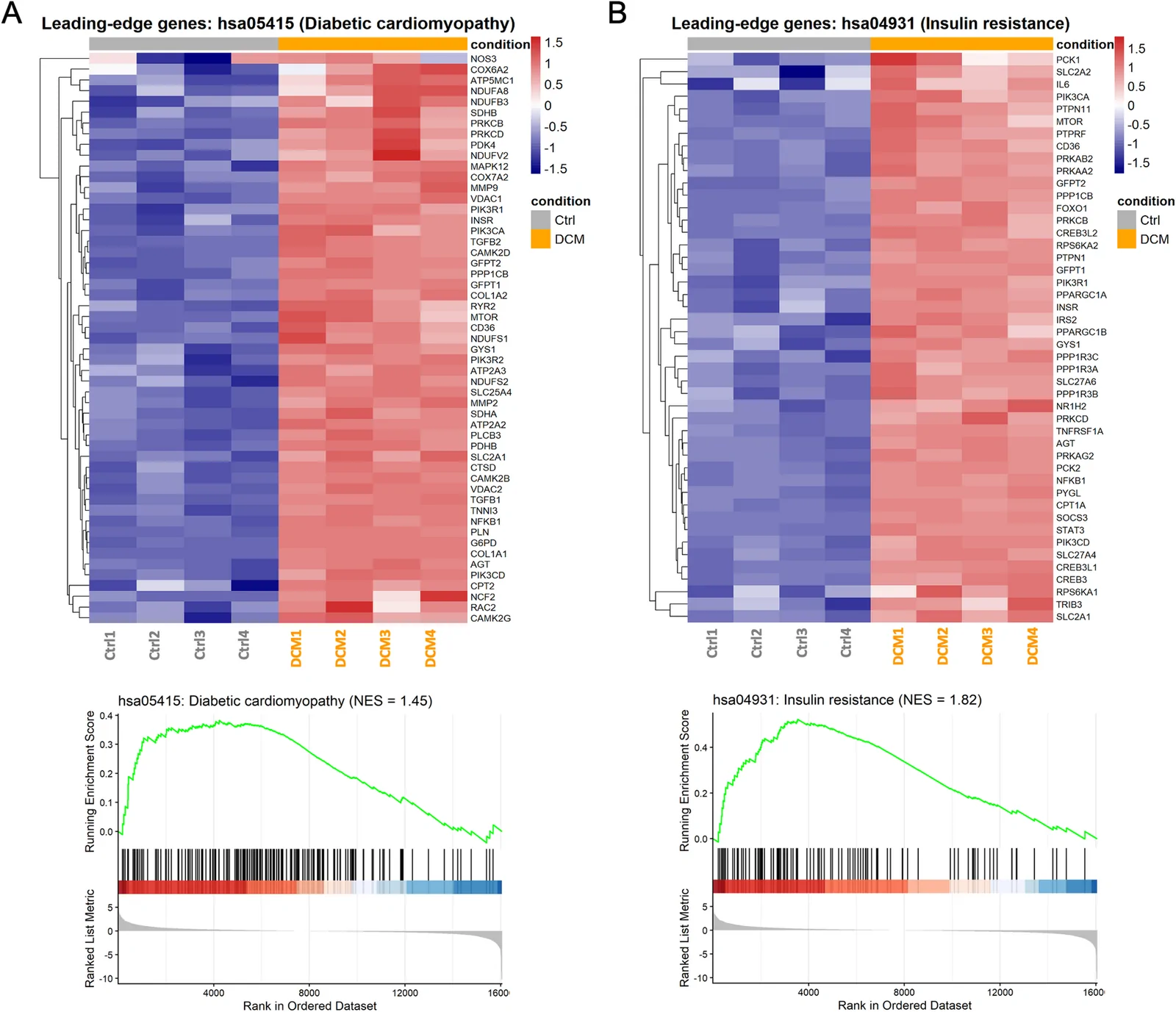
Gene set enrichment analysis (GSEA) of diabetic cardiomyopathy and insulin resistance pathways. Heatmaps display leading-edge genes contributing to pathway enrichment, with row-scaled VST-normalised expression across control (grey) and DCM (diabetic cardiomyopathy, orange) samples. **A** Diabetic cardiomyopathy pathway (KEGG: hsa05415) showing coordinated upregulation of disease-associated genes in DCM. **B** Insulin resistance pathway (KEGG: hsa04931) demonstrating consistent upregulation of key metabolic and inflammatory regulators. Corresponding enrichment plots are shown below each heatmap (normalized enrichment score, NES)

**Fig. 4:**
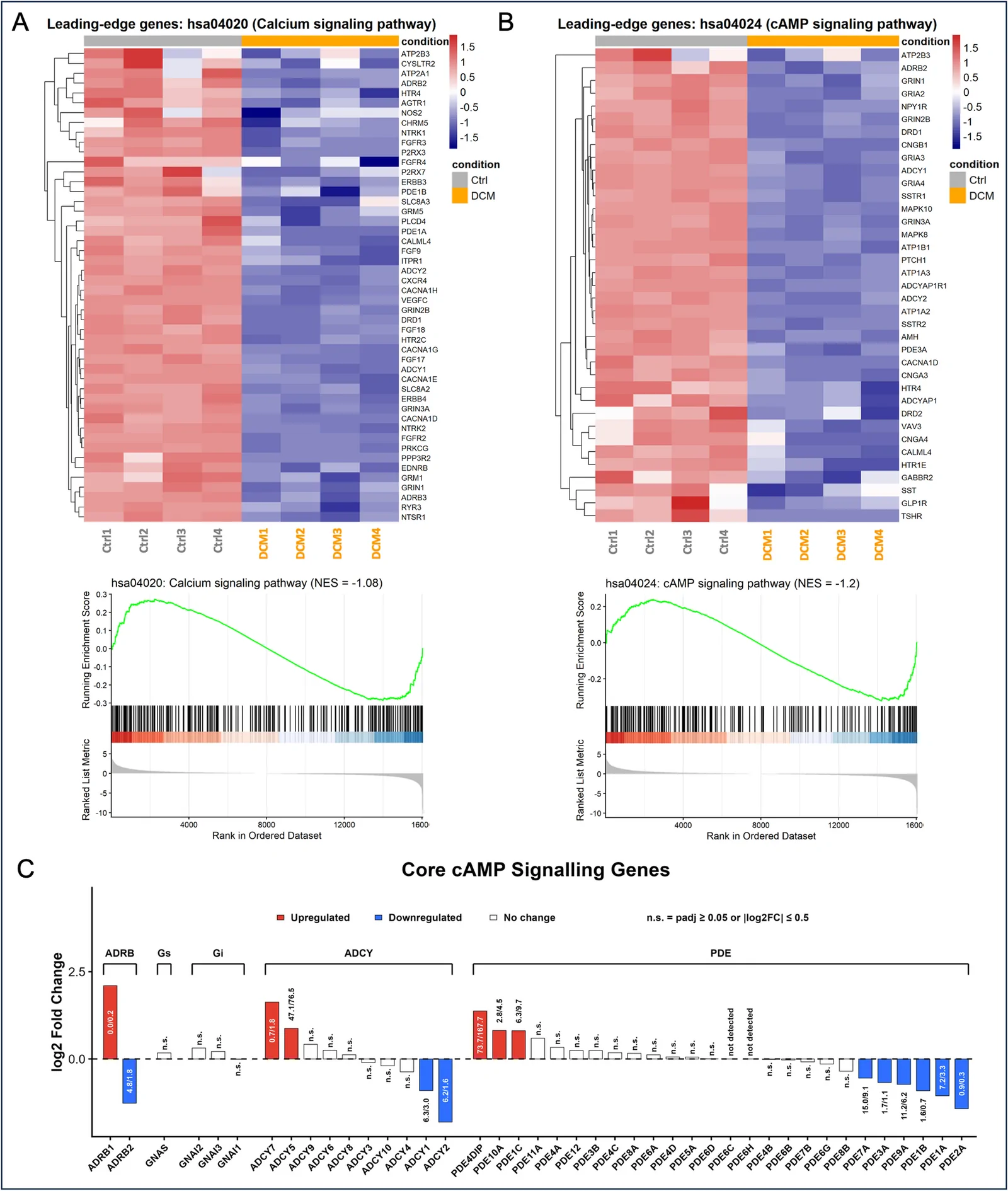
Extension of pathway-level analysis highlighting suppressed calcium signaling and cyclic nucleotide in diabetic cardiomyopathy (DCM). **A** Calcium signaling pathway (KEGG: hsa04020) and **B** cAMP signaling pathway (KEGG: hsa04024) show negative enrichment, with leading-edge gene heatmaps demonstrating coordinated downregulation in DCM relative to controls. Corresponding enrichment plots are shown below each heatmap (NES). **C** Log2 fold changes of core cAMP-signalling genes, including β-adrenoceptors (ADRB), Gs and Gi proteins, adenylyl cyclases (ADCYs), and phosphodiesterases (PDEs). Numbers on significant bars are mean FPKM (fragments per kilobase of transcript per million mapped reads) values, shown as Control/DCM

Gene set enrichment analysis identified significant enrichment of diabetic cardiomyopathy–related signatures (NES = + 1.45), with leading-edge genes visualised by heatmap revealing coordinated upregulation of disease-associated markers under diabetic conditions (Fig. 3A). These included pathways associated with metabolic remodeling (*PDK4*,* CD36*,* GFPT2*), mitochondrial and oxidative stress signaling (*RAC2*,* NCF2*,* G6PD*,* COX6A2*), inflammatory and profibrotic remodeling (*MMP9*,* TGFB1*,* COL1A1*,* AGT*), and altered calcium–contractile regulation (*PLN*,* TNNI3*,* PIK3CD*). To further characterise these changes, three key signaling pathways were examined. Insulin resistance signaling showed strong positive enrichment (NES = + 1.82) (Fig. 3B), with leading-edge genes demonstrating coordinated upregulation of mediators including *CD36*,* SOCS3*,* TNFRSF1A*,* NFKB1*,* STAT3* and *FOXO1*, consistent with activation of inflammatory *JAK/STAT* and *NF-κB* pathways that antagonise insulin receptor–AKT signaling in the diabetic heart. In contrast, calcium signaling exhibited negative enrichment (NES = − 1.08), with leading-edge genes including ADRB2, ADCY1, ADCY2, ITPR1 and CACNA1D, consistent with impaired GPCR-linked calcium signalling in DCM (Fig. 4A). Similarly, cAMP signaling was negatively enriched (NES = − 1.2), with reduced expression of key components including G protein-coupled receptors such as *ADRB2* and adenylate cyclases including *ADCY2*, consistent with impaired cAMP signaling (Fig. 4B). Given the central role of cyclic nucleotide signaling in cardiac function, phosphodiesterase genes were examined in greater detail, with the top differentially expressed candidates shown in Fig. 4C. Among these, *PDE2A* exhibited the greatest downregulation and *PDE4DIP* the greatest upregulation. Spatial representations of these transcriptional changes within KEGG networks are shown in Fig. S5A–D, illustrating activation of diabetic cardiomyopathy and insulin resistance signaling, and coordinated suppression of cAMP- and calcium-linked components. Together, these transcriptional and pathway-level alterations demonstrate coordinated suppression of calcium- and cAMP-linked signaling programmes, while supporting that the iPSC-derived model recapitulates key molecular features of the diabetic cardiac state and is relevant for modelling diabetic cardiomyopathy.

### FRET-based cAMP imaging demonstrates impaired compartment-specific cAMP signaling reserve and recovery with FGF1

**Fig. 5:**
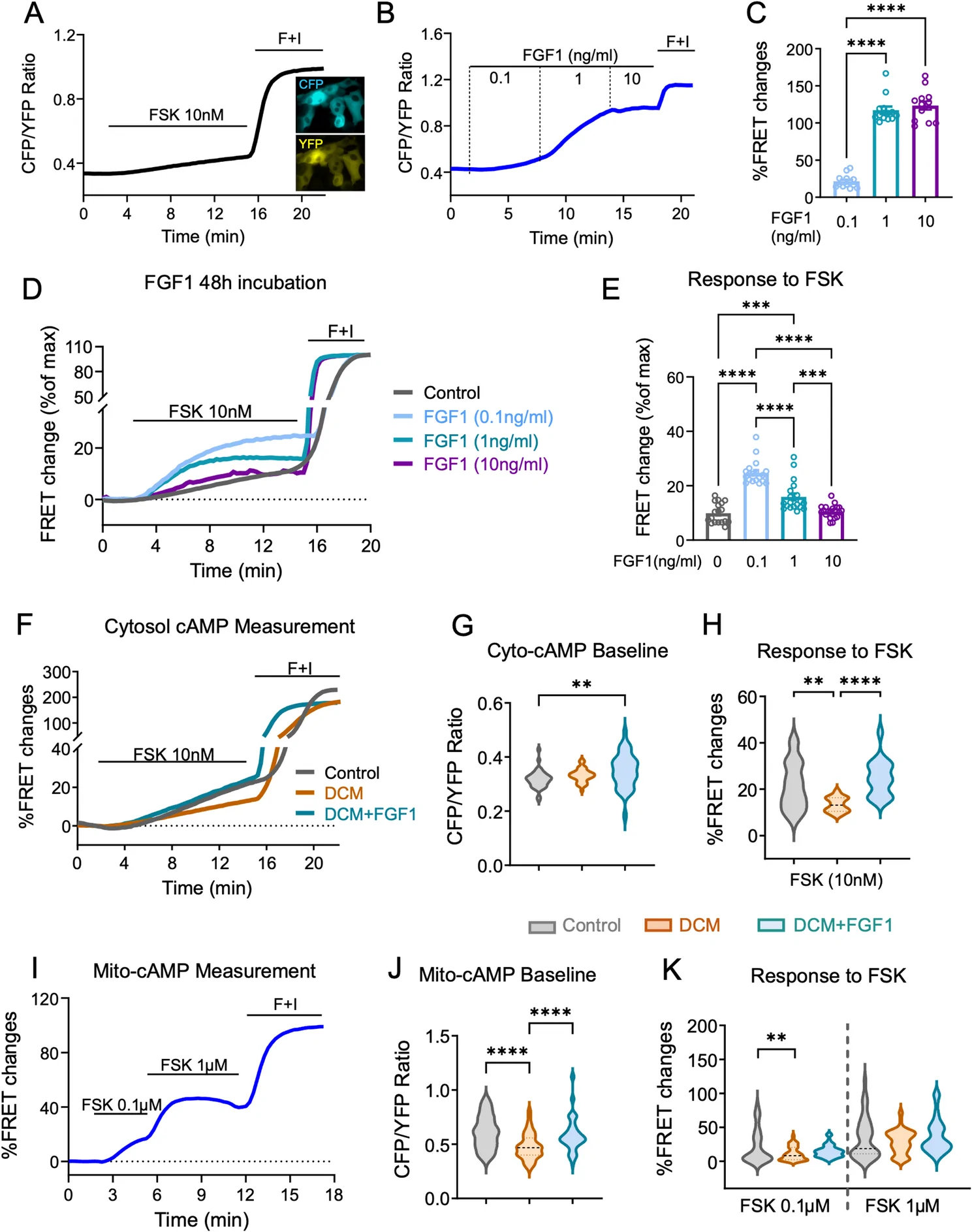
FGF1 modulates cytosolic and mitochondrial cAMP signaling in hiPSC-CMs. **A** Representative FRET traces (CFP/YFP ratio) showing cytosolic cAMP increase following forskolin (FSK, 10 nM) stimulation and maximal response after FSK (25 µM) and IBMX (100 µM). Insets show CFP and YFP fluorescence images of the biosensor. **B**, **C** Acute FGF1 stimulation (0.1–10 ng/ml) induces a dose-dependent increase in cAMP levels quantified as %FRET change (*n* = 13 cells, from a single differentiation batch). **D**,** E** After 48 h FGF1 incubation, FSK (10 nM)-evoked cAMP responses are enhanced in a concentration-dependent manner (control: *n* = 18, FGF1 0.1 ng/ml: *n* = 19, FGF1 1 ng/ml: *n* = 19, FGF1 10 ng/ml: *n* = 21, cells per group from a single differentiation batch). **F**–**H** Cytosolic cAMP measurements reveal preserved baseline (control: *n* = 40, DCM: *n* = 40, DCM+FGF1: *n* = 36 cells per group from two differentiation batches) but reduced FSK responsiveness in DCM, which is restored by 1 ng/ml FGF1 treatment (control: *n* = 20, DCM: *n* = 21, DCM+FGF1: *n* = 19 cells per group from two differentiation batches). **I**–**K** Mitochondrial cAMP measurements show reduced baseline (control: *n* = 45, DCM: *n* = 83, DCM+FGF1: *n* = 63 cells per group from two differentiation batches) and blunted responses to low-dose FSK (0.1 µM) in DCM, with partial recovery following 1 ng/ml FGF1 treatment (control: *n* = 45, DCM: *n* = 69, DCM+FGF1: *n* = 56 cells per group from two differentiation batches). *P < 0.05, **P < 0.01, ***P < 0.001, ****P < 0.0001; one-way ANOVA; n indicates the number of cells

cAMP–PKA signaling plays a central role in cardiomyocyte excitation–contraction coupling. To functionally assess alterations in this pathway suggested by bulk RNA sequencing, intracellular cAMP dynamics were examined across experimental groups using an Epac based fluorescence resonance energy transfer (FRET) sensor Epac-SH187. First, cardiomyocytes were stimulated with forskolin (FSK, 10 nM), a direct adenylyl cyclase activator, followed by a combination of forskolin and the phosphodiesterase inhibitor IBMX (F + I) to achieve maximal cAMP accumulation (Fig. 5A), confirmed that FSK at 10 nM was acceptable for a sufficient but sub-maximal FRET response and elucidated the time interval between drug additions to allow the response to plateau before the addition of further drug or saturation. To determine the optimal therapeutic dose, we then assessed FGF1 effect on cAMP dynamics in a dose-response manner (0.1, 1, and 10 ng/ml). All FGF1 concentrations significantly enhanced cAMP responses, with dose-dependent increases in FRET signals (Fig. 5B, C, ****p < 0.0001). Following 48 hour FGF1 pre-incubation, cardiomyocytes demonstrated enhanced cAMP generation capacity upon 10 nM FSK stimulation, with 0.1 and 1 ng/ml FGF1 treatment showing optimal enhancement (Fig. 5D, E), confirming FGF1’s ability to modulate the cAMP pathway. Based on these results, 1 ng/ml FGF1 was chosen for subsequent treatment of the DCM model.

We then compare cytosolic cAMP concentration in DCM model and assessed the effects of 48-hour pre-incubation with 1 ng/ml FGF1 on DCM cardiomyocytes. (Fig. 5F). Baseline CFP/YFP ratios were not changed in the DCM model when compare with control, and FGF1 treated DCM cells showed increased baseline cAMP levels (Fig. 5G, **p < 0.01). Upon 10 nM FSK stimulation, DCM cardiomyocytes exhibited markedly blunted cAMP responses compared to controls (Fig. 5H, **p < 0.01). Importantly, FGF1 treatment fully restored FSK-stimulated cAMP generation in DCM cardiomyocytes, with responses comparable to or exceeding control levels (Fig. 5H, ****p < 0.0001). To assess mitochondrial cAMP dynamics, we used an outer mitochondrial membrane (OMM)-targeted FRET sensor, OMM-H187, to monitor mitochondrial cAMP (mito-cAMP) changes (Fig. 5I). Notably, baseline mito-cAMP levels were significantly reduced in DCM cardiomyocytes compared to controls (Fig. 5J, ****p < 0.0001), contrasting with the unchanged cytosolic baseline. FGF1 treatment normalized this pathologically reduced baseline mito-cAMP toward control levels (Fig. 5J). Assessment of mito-cAMP responses to FSK stimulation (0.1 µM and 1 µM) showed that DCM cardiomyocytes had a reduced mito-cAMP increase, most evident at 0.1 µM (Fig. 5K; *p* < 0.01). FGF1 treatment improved mito-cAMP responsiveness, producing a modest increase at 0.1 µM and a robust recovery at 1 µM, reaching control-level or higher responses (Fig. 5K). These findings indicate compartment-specific cAMP dysregulation in DCM. Basal cytosolic cAMP is largely preserved, whereas mitochondrial outer-membrane cAMP is reduced. This pattern is associated with increased PDE4DIP expression and reduced ADCY2 expression, suggesting enhanced local cAMP breakdown, though direct mechanistic validation would be required to establish causality. Importantly, both cytosolic and mitochondrial compartments show impaired forskolin-stimulated cAMP responses, indicating reduced cAMP signaling reserve. FGF1 improves cAMP signaling by lowering PDE4DIP expression, thereby relieving excessive cAMP hydrolysis and restoring stimulated cAMP responses in both compartments, including at the mitochondrial outer membrane.

### Morphological and metabolic characterisation of hiPSC-derived cardiomyocytes recapitulates key features of diabetic cardiomyopathy

**Fig. 6:**
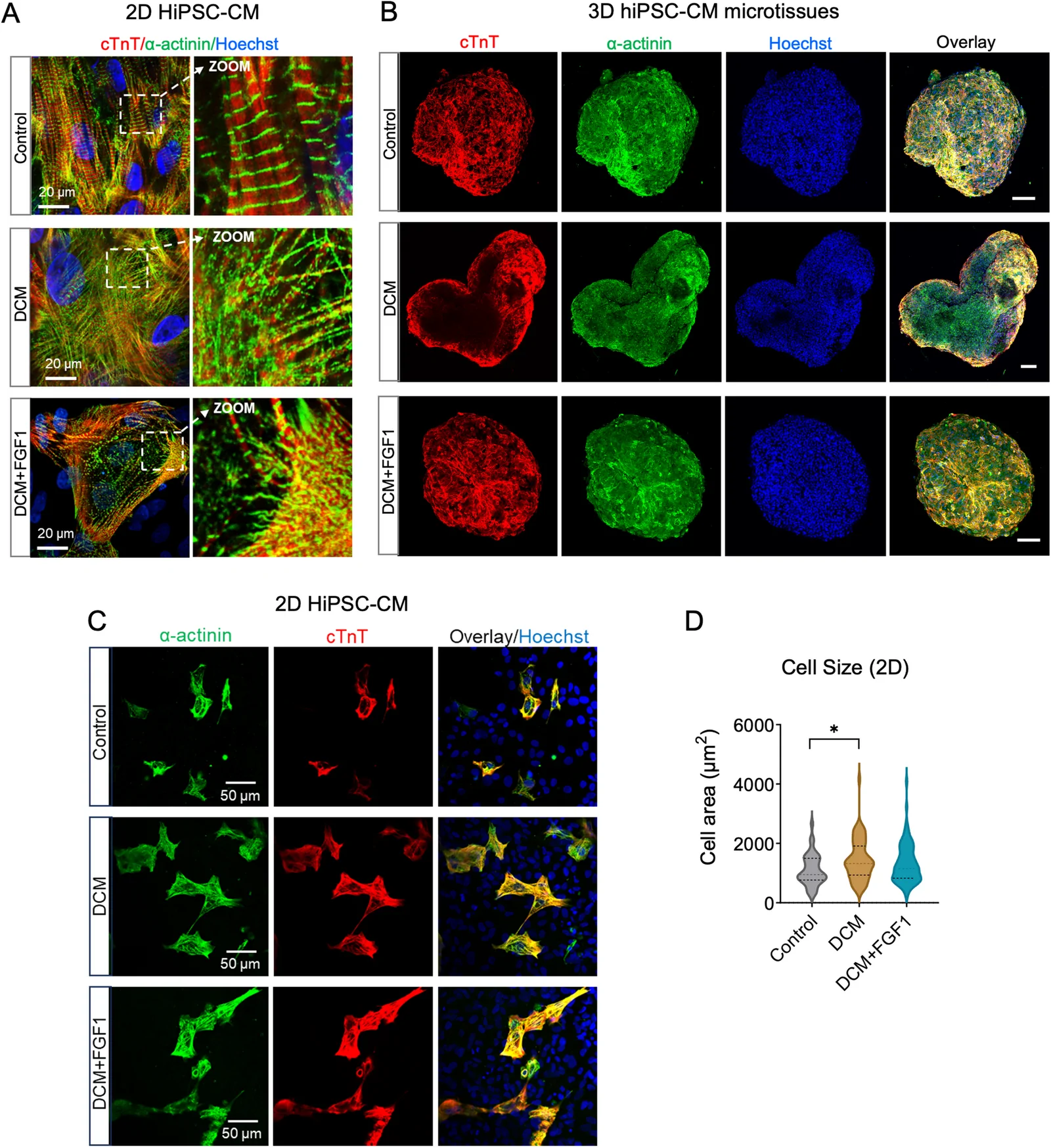
Structural remodeling of 2D and 3D hiPSC-CMs in DCM and rescue by FGF1. **A** Immunofluorescence staining of 2D hiPSC-CMs showing cTnT (red), α-actinin (green), and nuclei (Hoechst, blue). DCM cells display sarcomeric disorganization compared with control, which is partially restored by FGF1 treatment. Scale bar, 20 μm. **B** Representative images of 3D hiPSC-CM microtissues stained for cTnT, α-actinin, and Hoechst. DCM microtissues exhibit structural abnormalities that are improved with FGF1. Scale bar, 100 μm. **C**,** D** Lower-magnification 2D images used for cell area quantification (scale bar, 50 μm) and corresponding analysis showing increased cell size in DCM compared with control, which is attenuated by FGF1 treatment (control: *n* = 43, DCM: *n* = 65, DCM+FGF1: *n* = 92, cells per group from a single differentiation batch). Data are presented as mean ± SEM; *p < 0.05; One-way ANOVA; n indicates the number of cells

For the identification of morphological differences between control, diabetic cardiomyopathy (DCM), and FGF1-treated cardiomyocyte models, prepared 2D cultures and 3D microtissues were stained with immunofluorescence antibodies specific to cardiomyocytes. 2D hiPSC-derived cardiomyocytes were triple-stained with cardiac troponin T (cTnT, red), α-actinin (green), and Hoechst nuclear counterstain (blue) (Fig. 6A). Control cardiomyocytes displayed well-organized sarcomeric striations with clearly defined α-actinin bands, while DCM cardiomyocytes exhibited disrupted sarcomeric organization with less defined striations and altered cellular architecture. FGF1 treatment partially restored sarcomeric organization with improved α-actinin striation patterns compared to untreated DCM cardiomyocytes. 3D cardiac microtissues were stained for cTnT and α-actinin expression patterns (Fig. 6B). Limited by the variable size and three-dimensional architecture of these constructs, we failed to perform quantitative structural analysis. However, we observed that control microtissues exhibited uniform cTnT expression and well-distributed α-actinin staining, whereas DCM microtissues showed heterogeneous cTnT expression and disrupted α-actinin organization. FGF1-treated microtissues displayed intermediate characteristics with partially restored cTnT distribution and improved α-actinin organization. Nuclear staining revealed maintained cell viability across all conditions, indicating that structural changes occurred independently of cell death.

To quantitatively assess cellular hypertrophy, cardiomyocyte area (µm^2^) was measured in 2D cultures. Sarcomeric organisation was visualised using anti-cTnT and anti-α-actinin staining (Fig. 6C), which revealed organised sarcomeres across all conditions. An increased cell area was observed in DCM (1443.84 ± 85.42 µm^2^, *n* = 65) compared with control (10,96.81 ± 78.96 µm^2^, *n* = 43), consistent with a hypertrophic phenotype. Whereas the cardiomyocyte area in FGF1-treated models remained elevated (1317.32 ± 68.01 µm^2^, *n* = 92), this increase did not reach statistical significance compared to control (Fig. 6D).

### Metabolic characterisation of hiPSC-derived cardiomyocytes recapitulates key features of diabetic cardiomyopathy

**Fig. 7:**
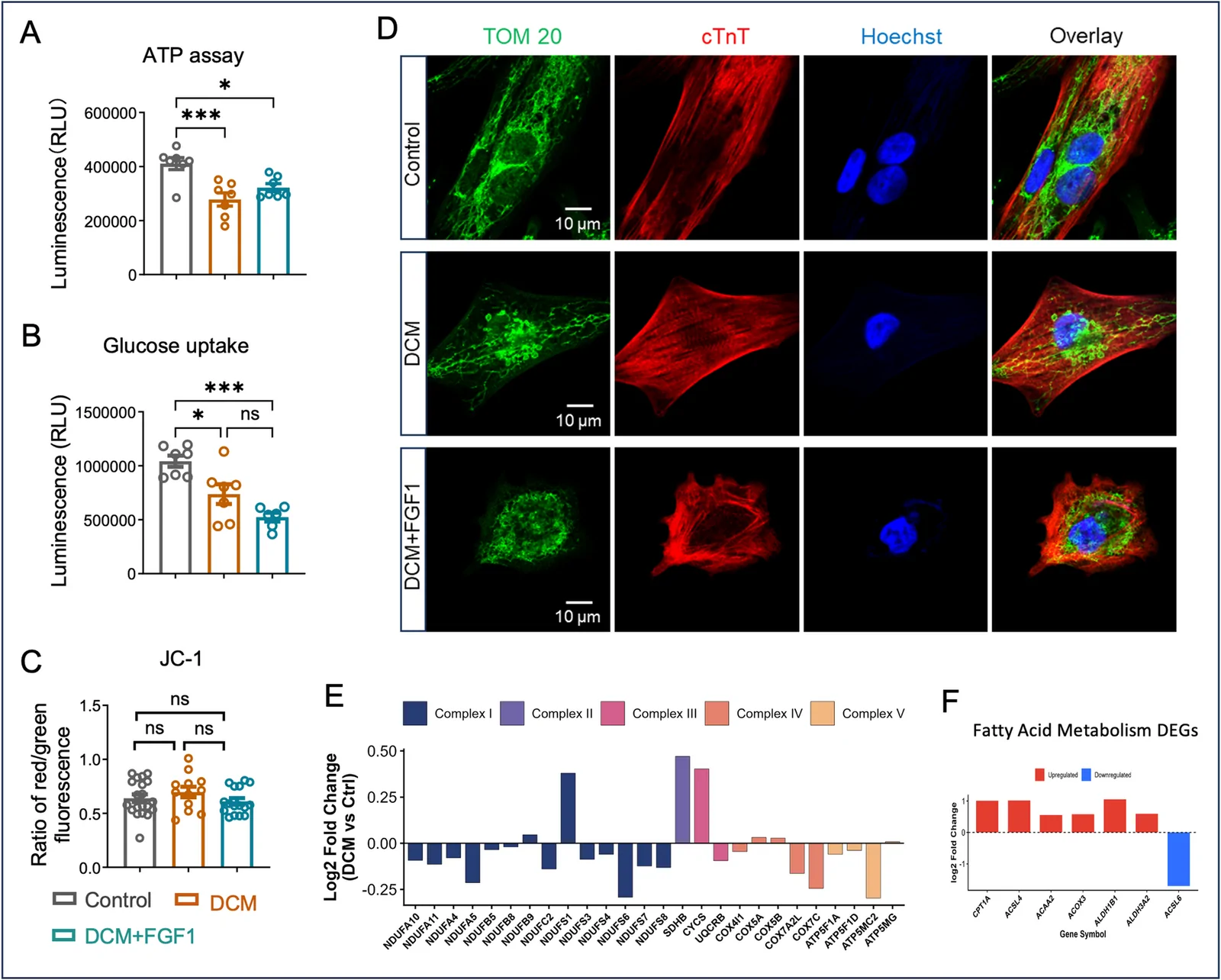
Metabolic and mitochondrial alterations in DCM hiPSC-CMs and modulation by FGF1. Intracellular ATP levels (*n* = 7 in each group) **A** and Glucose uptake **B** measured by luminescence-based assay (control: *n* = 7, DCM: *n* = 7, DCM+FGF1: *n* = 6); n indicates the number of wells. **C** JC-1 assay assessing mitochondrial membrane potential (red/green ratio; control: *n* = 27, DCM: *n* = 17, DCM+FGF1: *n* = 19); n indicates the number of cells. Data are mean ± SEM; *P < 0.05, ***P < 0.001, ns: not significant; one-way ANOVA. **D** Immunofluorescence staining for TOM20 (mitochondria, green), cTnT (red), and nuclei (Hoechst, blue). DCM cells display disrupted mitochondrial morphology, which is ameliorated by FGF1. Scale bar, 10 μm. Transcriptomic analysis shows dysregulation of oxidative phosphorylation genes, complexes I–V **E** and fatty acid metabolism related genes **F** in DCM, indicating impaired mitochondrial energy metabolism. Panels E and F are based on RNA-seq analysis of control and DCM groups only (from 4 independent pooled RNA samples per group, prepared from a single differentiation batch)

To examine metabolic function, we first performed a luminescence-based ATP assay to quantify cellular ATP as an indicator of viable, metabolically active cells (Fig. 7A). The DCM model exhibited a significant reduction in ATP production relative to control (2,78,125 ± 24,303, *n* = 7 vs. 4,10,692 ± 22,516, *n* = 7, *p* < 0.001). FGF1 treatment resulted in a partial recovery of ATP that remained statistically lower than control levels (3,21,704 ± 14,640, *n* = 7, *p* < 0.05 vs. control). Next, glucose uptake was measured under each condition (Fig. 7B). DCM cardiomyocytes showed reduced glucose uptake relative to control (7,37,583 ± 91,349, *n* = 7 vs. 10,42,653 ± 52,015, *n* = 7, *p* < 0.05), whereas the addition of FGF1 further decreased glucose uptake (5,23,473 ± 40,640, *n* = 6, *p* < 0.001 vs. control) in the diseased model. We used JC-1 staining to assess mitochondrial membrane potential (ΔΨm) in control, DCM, and FGF1-treated DCM cardiomyocytes (Fig. 7C). The ratio of red-to-green fluorescence, which reflects mitochondrial membrane polarization status, showed no significant differences between groups, indicating that mitochondrial membrane potential was preserved in the DCM model and remained unchanged following FGF1 treatment. Mitochondrial morphology was examined by immunostaining with anti-TOM20 (mitochondrial marker) and anti-cTnT (cardiomyocyte marker) (Fig. 7D). Control cardiomyocytes displayed elongated, well-organised, and parallel mitochondrial networks. In contrast, DCM cardiomyocytes exhibited smaller, rounded, and fragmented mitochondria with reduced structural organisation indicative of mitochondrial dysregulation. Treatment with FGF1 partially restored mitochondrial organisation towards a control-like phenotype.

Consistent with these functional and structural alterations, bulk RNA-sequencing analysis revealed comprehensive mitochondrial metabolic remodeling characteristic of DCM. Examination of electron transport chain components demonstrated complex-specific dysregulation (Fig. 7E), with predominant downregulation of Complex I subunits (except *NDUFS1*), while Complex II (*SDHB*) and Complex III (*CYCS*) genes were significantly upregulated, indicating compensatory mechanisms to maintain electron flow despite Complex I impairment. Complex IV and V genes showed variable expression patterns. Fatty acid metabolism genes demonstrated transcriptional signatures of metabolic remodeling associated with DCM (Fig. 7F), with marked upregulation of rate-limiting enzymes and transporters including *CPT1A*,* ACSL4*,* ACAA2*,* ACOX3*,* ALDH1B1*, and *ALDH3A2*, while *ACSL6* was reduced. This transcriptional pattern associated with DCM may indicate enhanced fatty acid oxidation capacity.

#### Validation of selected dysregulated genes

**Fig. 8:**
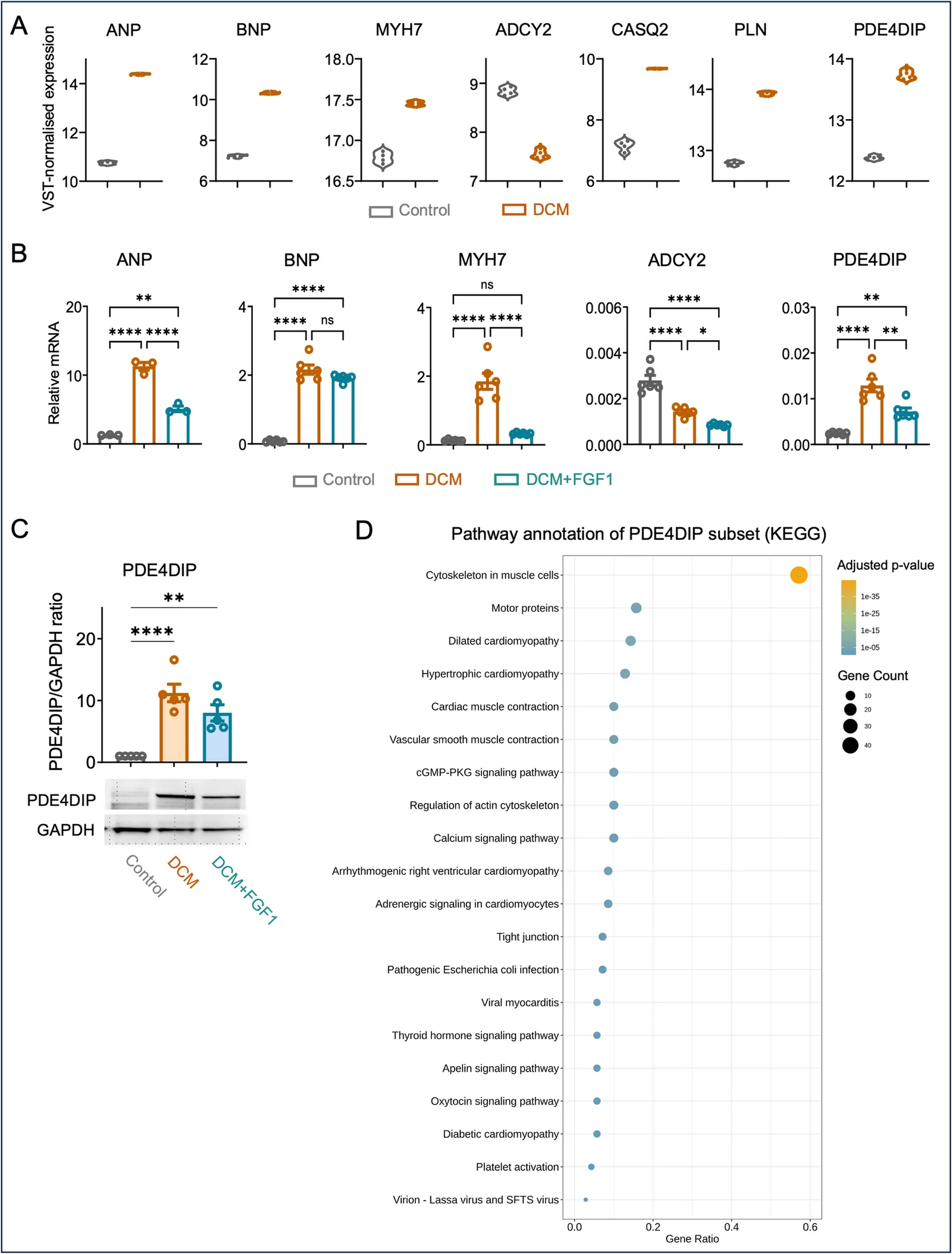
Transcriptomic dysregulation and molecular validation in the hiPSC-CM DCM model and response to FGF1. **A** RNA-seq–identified dysregulated cardiac dysfunction–related genes that were significantly dysregulated in DCM compared with control (4 independent pooled RNA samples per group, from a single differentiation batch). **B** qPCR validation of selected transcripts and modulation by FGF1 (6 independent pooled RNA samples per group, from 3 differentiation batches). **C** Western blotting confirming increased PDE4DIP in DCM, with attenuation following FGF1 treatment (GAPDH loading control; *n* = 6 independent pooled samples per group, from 3 independent differentiation batches). **D** KEGG pathway enrichment/annotation for the PDE4DIP-associated gene subset. Data are mean ± SEM. *P < 0.05, **P < 0.01, ***P < 0.001, ****P < 0.0001. ns, not significant; one-way ANOVA

RNA-seq analysis identified a focused set of cardiac dysfunction related transcripts that were significantly dysregulated in DCM hiPSC-CMs compared with controls, including ANP (*NPPA*), BNP (*NPPB*), *MYH7*, *ADCY2*,* CASQ2*,* PLN*, and *PDE4DIP* (Fig. 8A). To validate these findings, we quantified a subset of targets by qPCR (*ANP/NPPA*,* BNP/NPPB*,* MYH7*, *ADCY2*, and *PDE4DIP*), which reproduced the RNA-seq–derived direction of change in DCM and showed partial normalization with FGF1 treatment (Fig. 8B). Consistent with the mRNA data, western blot analysis further validated DCM-associated changes at the protein level and demonstrated attenuation of these alterations following FGF1 treatment (Figs. 8C, S6). These findings collectively suggest that FGF1 has the potential to ameliorate aspects of the diabetic cardiomyopathy phenotype by reducing the expression of key markers of cardiac dysfunction. Additionally, KEGG pathway analysis of the PDE4DIP-associated gene subset revealed a predominant enrichment of cardiomyopathy and muscle contraction pathways, including dilated cardiomyopathy, hypertrophic cardiomyopathy, arrhythmogenic right ventricular cardiomyopathy and cardiac muscle contraction, as well as calcium and adrenergic signaling in cardiomyocytes (Fig. 8D).

### Microelectrode array analysis reveals electrophysiological dysfunction in diabetic cardiomyopathy

**Fig. 9:**
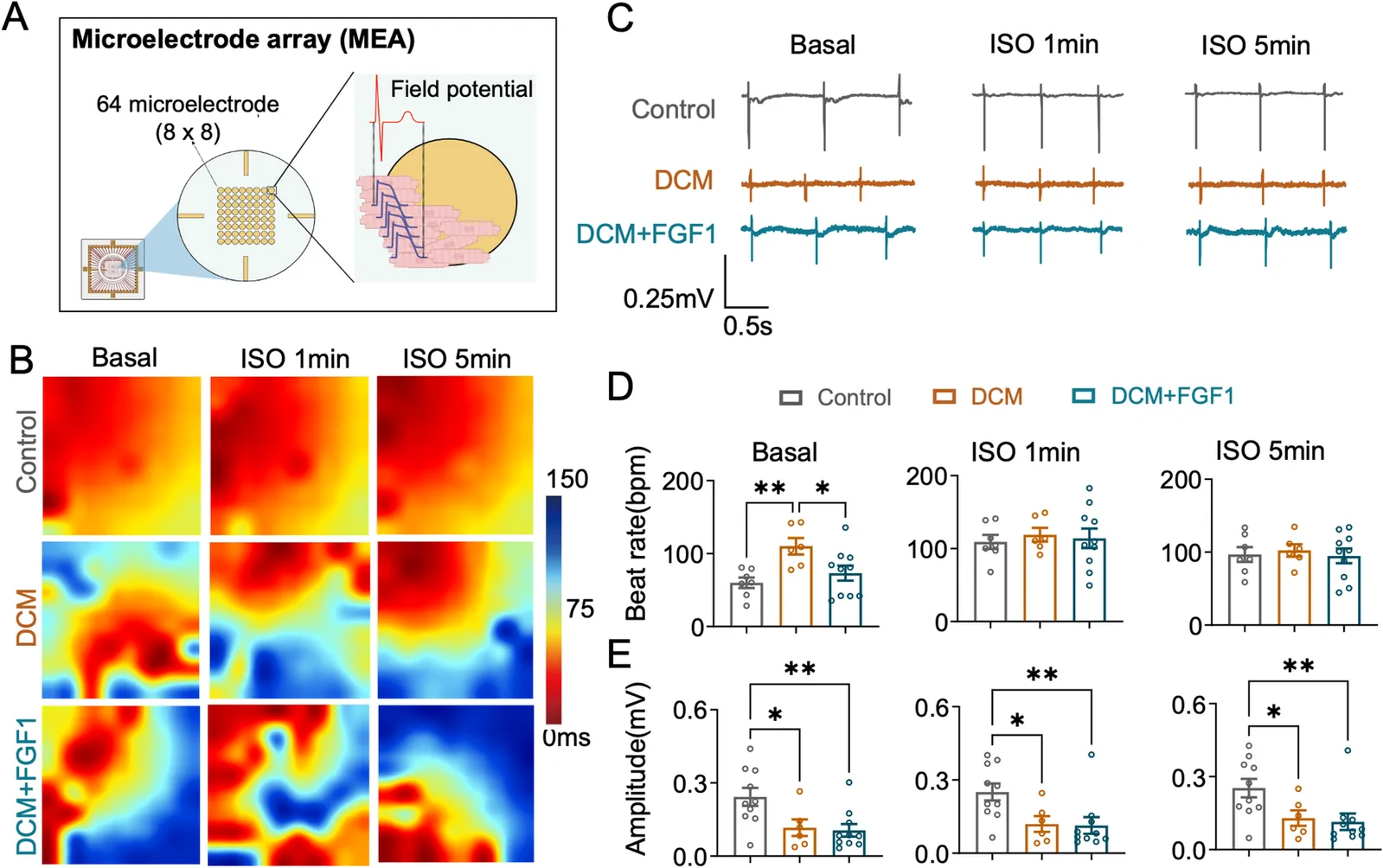
Microelectrode array (MEA) electrophysiological assessment of 2D hiPSC-derived cardiomyocytes (CM). **A** Schematic of 64-channel MEA recording system. **B** Representative activation maps at baseline and following isoproterenol (ISO; 1 and 5 min) in hiPSC-CM control, DCM, and DCM+FGF1 groups. **C** Representative field potential traces from control, DCM, and DCM+FGF1 groups. Quantification of beat rate **D** and field potential amplitude **E** from control (*n* = 10), DCM (*n* = 6), and DCM+FGF1 (*n* = 9). Data are mean ± SEM; statistical significance indicated. *P < 0.05, **P < 0.01; one-way ANOVA; n represents the total number of MEA wells from 3 independent differentiation batches

To characterise electrophysiological properties of hiPSC-derived cardiomyocytes under base-line and β-adrenergic stimulated conditions, microelectrode array (MEA) recordings were per-formed on control, DCM, and FGF1-treated cultures (Fig. 9). MEA enables non-invasive, long-term monitoring of spontaneous electrical activity and field potential dynamics across 64 electrodes simultaneously (Fig. 9A). Spatiotemporal activation mapping revealed distinct patterns of electrical propagation across the three conditions (Fig. 9B), with DCM cardiomyocytes displaying altered conduction properties compared to control, which were not restored by FGF1 treatment. Figure 9C showed representative field potential traces demonstrating characteristic morphological differences between groups under baseline and isoproterenol-stimulated conditions. At baseline, DCM cardiomyocytes exhibited a significantly increased spontaneous beat rate compared with control cells (**p < 0.01), with FGF1 treatment showing partial reduction toward control levels (*p < 0.05, Fig. 9D). This hyperexcitability in DCM was accompanied by a marked reduction in field potential amplitude (*p < 0.05, Fig. 9E), which persisted in FGF1-treated cells. Upon β-adrenergic stimulation with isoproterenol (ISO), beat rates increased across all groups, with no significant differences observed between conditions at either 1- or 5-minutes post-stimulation, indicating preserved chronotropic responsiveness (Fig. 9D). However, field potential amplitude remained significantly reduced in both DCM and DCM+FGF1 groups compared to control throughout the stimulation protocol (*p < 0.05 to **p < 0.01, Fig. 9E), suggesting persistent electrical remodeling that was not fully rescued by FGF1 treatment under the conditions tested. In contrast, repolarisation parameters: field potential duration (FPD), T-peak to T-end interval (Tpe), and QRS interval (QRSi) were not significantly different between DCM, DCM+FGF1, and control groups at any time point (Fig. S1), indicating that DCM and FGF1 treatment do not significantly affect repolarisation kinetics or conduction velocity under these conditions. Notably, 48 h FGF1 treatment alone only increased spontaneous beat rate at baseline compared with untreated controls, without affecting amplitude, FPD, Tpe, or QRSi (Fig. S2), suggesting a selective chronotropic effect that is independent of broader electrophysiological remodeling.

Taken together, these results demonstrate that DCM cardiomyocytes exhibit altered electrophysiological properties characterised by increased spontaneous beating and reduced field potential amplitude at baseline. While FGF1 treatment partially normalized the elevated baseline beat rate, it did not fully restore field potential amplitude.

### 3D optical mapping reveals impaired calcium handling in diabetic cardiomyopathy and partial FGF1-mediated rescue

**Fig. 10:**
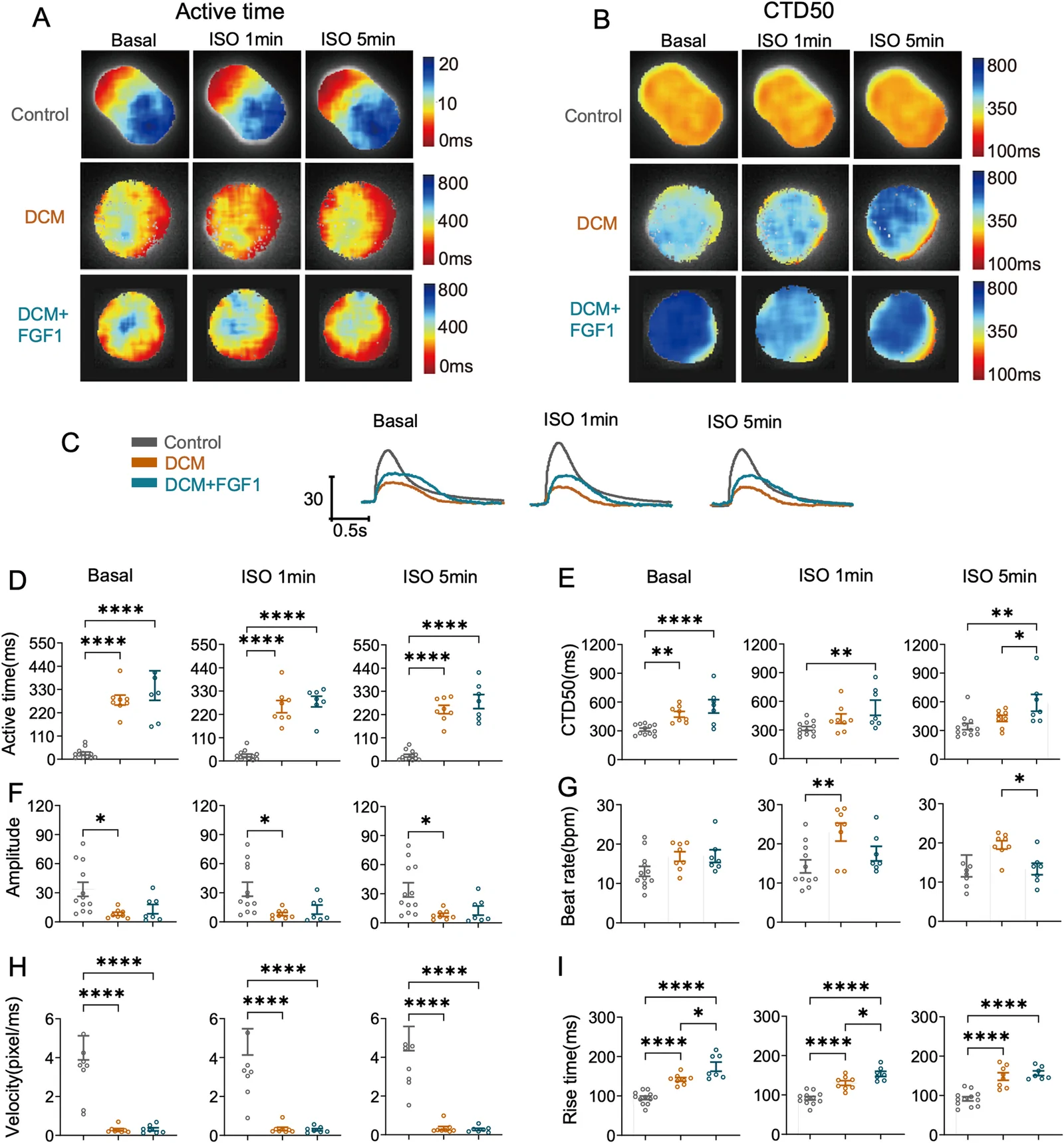
Optical mapping of calcium dynamics in 3D cardiac microtissues. **A**,** B** Spatial maps of activation time (active time) and calcium transient duration at 50% decay (CTD50) in control, DCM, and DCM+FGF1 microtissues at basal and following isoproterenol (ISO) stimulation (1 and 5 min). **C** Representative calcium transient traces from Control, DCM, and DCM+FGF1 groups. **D**–**I** Quantification of active time, calcium transient duration at 50% decay (CTD50), transient amplitude, beat rate, calcium propagation velocity, and rise time. DCM tissues exhibit prolonged activation and calcium kinetics, reduced amplitude and calcium propagation velocity, and impaired ISO responsiveness, which are partially improved by FGF1 treatment (control: *n* = 12, DCM: *n* = 8, DCM+FGF1: *n* = 7). Data are mean ± SEM; *P < 0.05, **P < 0.01, ***P < 0.001, ****P < 0.0001; one-way ANOVA; n represents the total number of 3D microtissues from 3 independent differentiation batches

3D cardiac microtissues provide a more physiologically relevant platform for studying the disease biology compared to traditional 2D culture, offering better recapturing of in vivo cellular microenvironment and intercellular coupling. Here, droplet printing technology was employed to fabricate 3D functional cardiac microtissues from hiPSC-derived cardiomyocytes for optical mapping analysis. To first establish the electrophysiological baseline of FGF1-treated healthy microtissues, optical mapping under 1 Hz field stimulation revealed that 48 h FGF1 treatment selectively enhanced activation time and calcium propagation velocity in control hiPSC-CM microtissues, without affecting calcium transient properties (Fig. S3), suggesting that FGF1 exerts a modest pro-conduction effect in the absence of disease. Spatiotemporal activation mapping during spontaneous beating revealed prolonged activation times in DCM microtissues compared to the synchronized, uniform activation observed in controls (Fig. 10A). Active time, representing the duration of electrical activation across the tissue, was significantly increased in DCM at baseline and remained elevated following β-adrenergic stimulation with isoproterenol, with no improvement observed after FGF1 treatment (****p < 0.0001, Fig. 10D). Calcium transient duration at 50% decay (CTD50) was markedly prolonged in DCM tissues at baseline (Fig. 10B), indicating impaired sarcoplasmic reticulum calcium reuptake. Interestingly, this difference diminished following isoproterenol stimulation, with DCM approaching control levels. FGF1 treatment significantly increased CTD50 across all conditions (Fig. 10E, ****p < 0.0001 at baseline; **p < 0.01 at ISO).

Representative calcium transient traces illustrated characteristic amplitude and kinetic differences between groups (Fig. 10C). Calcium transient amplitude was severely reduced in DCM across all conditions (*p < 0.05) and modestly improved by FGF1 treatment (Fig. 10F), suggesting partial improvement in calcium release from the sarcoplasmic reticulum. Spontaneous beat rate showed a complex pattern distinct from 2D MEA findings (Fig. 10G). At baseline, DCM showed no significant difference from control. However, at 1-minute post-isoproterenol, DCM exhibited markedly elevated beat rate (**p < 0.01), indicating exaggerated β-adrenergic responsiveness. By 5 min, this difference was attenuated. This divergence from MEA results likely reflects differences between 2D monolayer and 3D microtissue architectures, where enhanced mechanical coupling may modulate automaticity differently. Calcium propagation velocity was dramatically impaired in DCM (****p < 0.0001) and remained significantly reduced in both DCM and FGF1-treated groups (Fig. 10H), indicating persistent structural remodeling. Calcium rise time was significantly prolonged in DCM and further increased following FGF1 treatment at baseline and ISO 1 min (*p < 0.05 to ****p < 0.0001) (Fig. 10I). Optical mapping of calcium transients was additionally performed in 3D cardiac microtissues from an independent healthy WT hiPSC line (Fig. S4).

These 3D optical mapping data demonstrate that DCM involves severe calcium handling dysfunction, including reduced amplitude, prolonged cycling, impaired conduction, and altered β-adrenergic responsiveness. FGF1 achieved partial rescue by improving calcium transient amplitude, though paradoxically prolonged CTD50 and rise time. The divergent chronotropic responses between 2D and 3D models highlight the importance of dimensionality in disease modelling and indicate that cAMP pathway restoration primarily addresses calcium release amplitude but not temporal dynamics or conduction abnormalities.

## Discussion

This study identifies compartment-specific cAMP dysregulation as a key feature associated with diabetic cardiomyopathy, with ADCY2 downregulation and PDE4DIP upregulation differentially affecting cytosolic and mitochondrial cAMP pools. Using integrated 2D and 3D hiPSC-derived cardiac models, we reveal that FGF1 restores cAMP signaling and partially improves calcium handling but leaves structural conduction defects unresolved, indicating distinct therapeutic targets for functional versus structural DCM abnormalities.

### DCM model validation and transcriptomic characterization

We adopted a hiPSC-derived cardiomyocyte model from Drawnel et al. [12], which induces structural and functional abnormalities by exposing cells to a composite diabetic-stress condition (high glucose, endothelin-1, cortisol), and confirmed that our model recapitulated key DCM hallmarks including cellular hypertrophy, reduced viability, impaired glucose uptake, and transcriptional signatures of metabolic remodeling. While multiple studies using high glucose, lipid overload, or insulin-resistant conditions in hiPSC-CMs report convergent DCM features, comprehensive reviews emphasize that existing models capture only subsets of the diabetic phenotype [29, 30], a limitation we addressed by integrating complementary approaches where 2D cultures provided controlled environments for mechanistic cAMP pathway dissection and FRET-based compartment-specific measurements, while 3D cardiac microtissues enabled assessment of tissue-level calcium dynamics and conduction properties through optical mapping. Bulk RNA-sequencing validated our model’s disease relevance by confirming canonical DCM markers, significant enrichment of the KEGG DCM pathway (hsa05415), and transcriptional evidence of upregulated fatty acid oxidation machinery, demonstrated by robust upregulation of *PDK4*,* CPT1A*, and fatty acid β-oxidation enzymes coupled with Complex I impairment and compensatory Complex II (*SDHB*) and Complex III (*CYCS*) upregulation. These transcriptional signatures of metabolic remodeling reduce cardiac efficiency and increases oxidative stress. The association between impaired cAMP signaling and metabolic gene expression changes suggests a potential relationship, though direct causality would require further mechanistic validation.

### Compartment-specific cAMP dysfunction: a unifying mechanism for DCM pathogenesis

The identification of PDE4DIP as an important dysregulated scaffold protein represents a potentially significant mechanistic advance in understanding DCM progression. Our findings parallel recent work by Gu et al. [31] in left ventricular non-compaction, where PDE4DIP upregulation drove cytoskeletal dysfunction via Rho-ROCK signaling. In the diabetic context, however, concurrent *ADCY2* downregulation and *PDE4DIP* upregulation act convergently to deplete local cAMP -reduced synthesis and accelerated hydrolysis reinforce one another, selectively disrupting microdomain-specific PKA signaling that governs excitation-contraction coupling, sarcomeric organisation, and mitochondrial integrity. The resultant structural disarray is reflected in the heterogeneous cTnT expression, disrupted α-actinin organisation, and mitochondrial fragmentation observed in our DCM cardiomyocytes. This disease-context specificity of PDE4DIP dysfunction suggests it functions as a nodal integrator whose pathogenic effects are shaped by the underlying metabolic environment.

The compartment-specific nature of cAMP dysfunction provides new mechanistic insight into the differential vulnerability of cAMP microdomains in DCM. Cytosolic baseline cAMP was preserved, yet stimulated responses were significantly reduced, whereas mitochondrial pools exhibited both reduced resting cAMP and impaired stimulated responses. This dual-compartment pattern suggests that ADCY2 downregulation impairs stimulus-coupled cAMP generation globally, whilst PDE4DIP-anchored hydrolysis exerts a disproportionate effect on mitochondrial resting cAMP, rendering this compartment dually vulnerable. The selective collapse of mitochondrial baseline cAMP likely reflects PDE4DIP accumulation at mitochondria-associated membranes, where it may anchor PDE4D isoforms in proximity to the mitochondrial outer membrane, creating a zone of constitutive local cAMP hydrolysis that chronically suppresses mitochondrial cAMP–PKA signaling; one consequence may be reduced PKA-mediated phosphorylation of Drp1 at Ser637, a known inhibitory site whose loss promotes Drp1 translocation to the outer mitochondrial membrane and pathological fission [32, 33], consistent with the mitochondrial fragmentation observed in our DCM cardiomyocytes. This mechanistic framework may explain the complex clinical pattern of preserved resting cardiac function with disproportionately impaired stress responses observed in early diabetic cardiomyopathy [34]. In the present hiPSC-CM RNA-seq dataset, ADRB1 showed very low baseline expression compared with ADRB2, which may reflect the immature/foetal-like adrenergic profile of hiPSC-CMs. Therefore, ADRB2 and ADCY2 were highlighted as more robustly expressed cAMP-pathway components. However, receptor-specific β1- versus β2-adrenergic remodeling cannot be inferred from transcriptomic data alone and will require future validation using receptor-selective pharmacological approaches. Transcriptomic analysis confirmed characteristic metabolic gene expression changes in DCM, with upregulated fatty acid oxidation machinery (*PDK4*,* CPT1A*) and mitochondrial Complex I dysfunction, establishing our model as representative of human DCM pathophysiology.

Importantly, 2D MEA data indicate that chronotropic responsiveness to isoproterenol is preserved in DCM hiPSC-CMs, suggesting that β-adrenergic responsiveness is not abolished but that compartment-specific cAMP signaling reserve is impaired. This phenotype was not observed in 3D microtissues at baseline, suggesting that dimensionality and mechanical coupling influence automaticity. The elevated beating rate in 2D cultures may contribute to the increased arrhythmia susceptibility reported clinically in DCM patients, where enhanced automaticity could combine with compromised β-adrenergic reserve to create a proarrhythmic substrate [35]. Transcriptomic analysis also revealed upregulation of both *PLN* and *CASQ2* in DCM cardiomyocytes, providing a mechanistic link between cAMP microdomain dysfunction and impaired SR calcium handling. As a key PKA substrate, PLN normally undergoes Ser16 phosphorylation during β-adrenergic stimulation to relieve SERCA2a inhibition [36]; reduced cAMP–PKA signaling would therefore sustain constitutive SERCA2a inhibition and impair SR Ca^2+^ reuptake. Transcriptional upregulation of *PLN* itself further amplifies this inhibitory tone, compounding the phosphorylation deficit. Concurrently, upregulated *CASQ2* may paradoxically worsen RyR2-mediated Ca^2+^ leak despite its buffering role, prolonging Ca^2+^ transients and contributing to the proarrhythmic substrate. Together, these changes create a dual impairment of SR Ca^2+^ cycling: reduced refilling via SERCA2a and dysregulated release via RyR2, that likely underlies both the contractile dysfunction and enhanced automaticity observed in our DCM cardiomyocytes [37].

### Three-dimensional microtissues recapitulate tissue-level electrophysiological dysfunction

The transition from 2D cellular hyperexcitability to 3D tissue-level dysfunction represents a critical finding with direct clinical relevance [17]. While individual DCM cardiomyocytes exhibited enhanced automaticity in 2D culture, 3D cardiac microtissues displayed severely impaired calcium propagation velocity and heterogeneous calcium propagation closely mirror electrophysiological abnormalities in DCM patients [38]. This dimensionality-dependent divergence reflects emergent tissue-level properties not readily predictable from single-cell behaviour, underscoring the importance of 3D platforms for disease modelling [17].

Because 3D optical mapping was performed using the calcium indicator Rhod-2, the reported 3D velocity represents calcium transient propagation velocity, or apparent activation derived from calcium signals, rather than direct electrical conduction velocity. Therefore, comparisons with MEA-derived electrical parameters should be interpreted cautiously, as the two methods measure related but distinct aspects of excitation–calcium coupling in different culture architectures. Optical mapping of 3D microtissues revealed profound calcium handling dysfunction, including reduced transient amplitude, prolonged duration, and slowed upstroke velocity, consistent with the diastolic dysfunction and impaired contractile reserve characteristic of clinical DCM [39, 40]. As discussed above, these deficits are mechanistically anchored in *PLN*-mediated SERCA2a inhibition and *CASQ2*-associated RyR2 dysregulation downstream of reduced cAMP–PKA signaling, together impairing both SR Ca^2+^ reuptake and release.

Spatial heterogeneity revealed by optical mapping that non-uniform wavefront propagation and prolonged activation times, has important implications for DCM arrhythmogenesis. These findings suggest that metabolic dysfunction creates electrical substrate heterogeneity predisposing to arrhythmias, consistent with increased sudden cardiac death risk in DCM patients [41]. Notably, the persistence of conduction defects despite partial functional rescue by FGF1 treatment suggests that structural remodeling represents an additional therapeutic target beyond metabolic signaling alone, and that combination strategies will likely be required for meaningful clinical translation. A later-maturation WT hiPSC-CM batch (day 44, Fig. S4) showed a clearer isoproterenol-induced increase in beat rate and calcium transient amplitude in control microtissues, suggesting that 3D β-adrenergic responsiveness improves with maturation; however, this observation remains preliminary.

### FGF1 therapeutic mechanism and translational implications

FGF1 emerges as a promising therapeutic candidate that operates through complementary mechanisms: normalizing PDE4DIP expression to restore compartmentalised cAMP signaling, and modulating ERK/MAPK pathway activity in a manner that may further influence PDE4 isoform regulation [42]. This dual action likely underlies the pattern of partial but meaningful rescue observed: cAMP-dependent functional deficits were substantially corrected, whilst established structural remodeling persisted. consistent with the principle that signaling dysfunction is more amenable to pharmacological reversal than downstream architectural changes. The differential rescue pattern has direct therapeutic implications. FGF1 effectively improved calcium transient amplitude and normalized β-adrenergic chronotropic responses, suggesting potential benefits for contractile function and exercise tolerance. However, persistence of conduction velocity defects indicates that comprehensive DCM treatment will require combination strategies additionally targeting gap junction remodeling or extracellular matrix reorganisation.

Taken together, these findings position FGF1 as particularly suited for early-stage DCM intervention, where cAMP microdomain dysfunction precedes extensive structural remodeling. This mechanistic specificity—targeting upstream signaling architecture rather than downstream effectors—distinguishes FGF1 from conventional heart failure therapies and suggests that therapeutic window is an important consideration: earlier intervention may prevent progression to the irreversible structural changes that limit rescue in established disease.

### Limitations and future directions

Several limitations warrant acknowledgement. Because the day 44 WT validation data were obtained in a single batch, further replication across independent differentiations will be required. FGF1 monotherapy achieved partial rescue, indicating that combination approaches incorporating antifibrotic agents, gap junction modulators, or mitochondrial-targeted therapies will likely be required for comprehensive DCM reversal. In this context, FGF1ΔHBS—a heparin-binding-deficient FGF1 variant with reduced proliferative potency—represents a compelling refinement worth investigating, as it has been shown to prevent DCM by maintaining mitochondrial homeostasis and reducing oxidative stress via AMPK/Nur77 suppression [14], directly relevant to the mitochondrial cAMP dysfunction and fragmentation identified here. Validation in patient-derived hiPSC-CMs with defined diabetic genetic profiles will also be essential to assess translational robustness and inter-individual variability. Additionally, our model has metabolic validation limitations. While we demonstrate transcriptional signatures of insulin resistance and reduced glucose uptake, formal insulin sensitivity assessment (e.g., insulin-stimulated glucose uptake curves) was not performed. Furthermore, our metabolic remodeling claims are primarily based on transcriptional signatures rather than direct metabolic flux measurements. Future studies incorporating metabolic flux analysis (e.g., seahorse profiling) would provide stronger functional validation of the observed gene expression changes and definitively establish altered substrate utilization.

Mechanistic validation of PDE4DIP as a causal driver rather than a consequence of metabolic stress remains a priority. CRISPR-mediated knockout and dominant-negative PDE4DIP constructs delivered via viral transduction would directly test whether PDE4DIP upregulation is necessary and sufficient for compartment-specific cAMP dysfunction, providing the causal evidence required to advance it as a bona fide therapeutic target. Additionally, FGF1 likely acts through multiple pathways beyond PDE4DIP regulation, and the partial rescue pattern suggests that PDE4DIP normalization alone may not account for all of FGF1’s effects.

Although the hiPSC-CM differentiation protocol included lactate-based metabolic purification and has previously achieved cTnT-positive rates above 80% in laboratory quality-control experiments [17, 43], cardiomyocyte purity was not quantified by flow cytometry in the specific batches used in this study. Future studies should include flow cytometric quantification of cTnT-positive cells across differentiation batches. Despite this limitation, the integrated 2D–3D platform established here offers a scalable framework for evaluating candidate therapies across both cellular and tissue-level endpoints, a capability that proved essential for revealing conduction defects invisible in 2D assessment alone. Ultimately, translating the compartment-specific cAMP dysfunction paradigm into clinical practice will require non-invasive biomarkers capable of detecting microdomain-level signaling deficits at early disease stages, shifting the therapeutic goal from treating established cardiomyopathy to preventing irreversible cardiac remodeling in diabetic patients.

## Conclusion

These findings identify PDE4DIP as a potential therapeutic target and suggest that DCM contains both reversible and irreversible signaling components coupled to cAMP alongside structural changes that resist pharmacological correction, though additional mechanistic validation would strengthen the therapeutic rationale. Together, they suggest that early intervention targeting the cAMP pathway integrity may prevent progression to irreversible structural remodeling in diabetic hearts.

## Supplementary Information

Below is the link to the electronic supplementary material.

Supplementary Material 1.

## Data Availability

No datasets were generated or analysed during the current study.

## Abbreviations

2D: Two-dimensional
2-DG: 2-Deoxyglucose
3D: Three-dimensional
ADCY2: Adenylyl cyclase 2
ANOVA: Analysis of variance
APOA4: Apolipoprotein A-IV
ATP: Adenosine triphosphate
BNP (NPPB): B-type natriuretic peptide
cAMP: Cyclic adenosine monophosphate
CASQ2: Calsequestrin 2
cDNA: Complementary DNA
CFP/YFP: Cyan fluorescent protein/yellow fluorescent protein
CLDN5: Claudin 5
cTnT: Cardiac troponin T
CTD50: Calcium transient duration at 50% decay
DCM: Diabetic cardiomyopathy
DESeq2: Differential expression sequencing 2
DMEM: Dulbecco’s modified eagle medium
EGFL6: EGF like domain multiple 6
Epac: Exchange protein directly activated by cAMP
FGF1: Fibroblast growth factor 1
FDR: False discovery rate
FRET: Förster resonance energy transfer
FSK: Forskolin
GAPDH: Glyceraldehyde 3-phosphate dehydrogenase
GSEA: Gene set enrichment analysis
GO: Gene ontology
HEPES: 4-(2-hydroxyethyl)-1-piperazineethanesulfonic acid
hiPSC: Human induced pluripotent stem cell
hiPSC-CM: hiPSC-derived cardiomyocyte
HRP: Horseradish peroxidase
IBMX: 3-isobutyl-1-methylxanthine
ISO: Isoproterenol
ITS-G: Insulin-transferrin-selenium
JC-1: 5,5ʹ,6,6ʹ -Tetrachloro-1,1ʹ,3,3ʹ-tetraethylbenzimidazolylcarbocyanine iodide
KEGG: Kyoto encyclopedia of genes and genomes
MEA: Microelectrode array
MEM: Minimum essential medium
MSigDB: Molecular signatures database
MYH7: Myosin heavy chain 7
NES: Normalized enrichment score
NPPA (ANP): Atrial natriuretic peptide
OMM: Outer mitochondrial membrane
PBS: Phosphate buffered saline
PCA: Principal component analysis
PDE4DIP: Phosphodiesterase 4D interacting protein
PKA: Protein kinase A
PLN: Phospholamban
PVDF: Polyvinylidene fluoride
qPCR: Quantitative polymerase chain reaction
RNA-seq: RNA sequencing
RLU: Relative light units
RIPA: Radioimmunoprecipitation assay buffer
SEM: Standard error of the mean
SPP1: Secreted phosphoprotein 1
TOM20: Translocase of outer mitochondrial membrane 20
TTR: Transthyretin
VST: Variance-stabilising transformation
WT: Wild type
∆Ψm: Mitochondrial membrane potential

## References

[1] Saeedi P, Petersohn I, Salpea P, Malanda B, Karuranga S, Unwin N, et al. Global and regional diabetes prevalence estimates for 2019 and projections for 2030 and 2045: results from the international diabetes federation diabetes atlas, 9th edition. Diabetes Res Clin Pract. 2019;157:107843. 10.1016/j.diabres.2019.107843. 31518657

[2] Kenny HC, Abel ED. Heart failure in type 2 diabetes mellitus. Circul Res. 2019;124:121–41. 10.1161/CIRCRESAHA.118.311371. PMC6447311 30605420

[3] Dillmann WH. Diabetic cardiomyopathy. Circul Res. 2019;124:1160–2. 10.1161/CIRCRESAHA.118.314665. PMC6578576 30973809

[4] Honka H, Solis-Herrera C, Triplitt C, Norton L, Butler J, DeFronzo RA. Therapeutic manipulation of myocardial metabolism: JACC state-of-the-art review. J Am Coll Cardiol. 2021;77:2022–39. 10.1016/j.jacc.2021.02.057. PMC8091273 33888253

[5] Guan X, Zhou C, Zhuo X, Yang W, Wang H. The heart of diabetes: unraveling metabolic drivers of cardiomyopathy. Cardiovasc Diabetol Endocrinol Rep. 2025;11:18. 10.1186/s40842-025-00231-x. PMC12416072 41013814

[6] Zaccolo M, Kovanich D. Nanodomain cAMP signalling in cardiac pathophysiology: potential for developing targeted therapeutic interventions. Physiol Rev. 2025;105:541–91. 10.1152/physrev.00013.2024. PMC7617275 39115424

[7] Xiang YK. Compartmentalization of β-adrenergic signals in cardiomyocytes. Circul Res. 2011;109:231–44. 10.1161/CIRCRESAHA.110.231340. PMC3493619 21737818

[8] Li D, Liu K, Davis H, Robertson C, Neely OC, Tarafdar A, et al. Abnormal cyclic nucleotide signaling at the outer mitochondrial membrane in sympathetic neurons during the early stages of hypertension. Hypertension. 2022;79:1374–84. 10.1161/HYPERTENSIONAHA.121.18882. PMC9172895 35506379

[9] Tenner B, Getz M, Ross B, Ohadi D, Bohrer CH, Greenwald E et al. Spatially compartmentalized phase regulation of a Ca^2+^-cAMP-PKA oscillatory circuit. Mayor S, Cooper JA, Nadler A (eds). eLife. 2020;9:e55013. 10.7554/eLife.55013 PMC7671691 33201801

[10] Aslam M, Ladilov Y. Regulation of mitochondrial homeostasis by sAC-derived cAMP pool: basic and translational aspects. Cells. 2021 [cited 2026 Mar 14];10. 10.3390/cells10020473. PMC7926680 33671810

[11] Chaoul V, Hanna R, Hachem P, El Hayek MS, Nour-Eldine W, Abou-Khalil P, et al. Differential changes in cyclic adenosine 3′-5′ monophosphate (cAMP) effectors and major Ca^2+^ handling proteins during diabetic cardiomyopathy. J Cell Mol Med. 2023;27:1277–89. 10.1111/jcmm.17733. PMC10148055 36967707

[12] Drawnel FM, Boccardo S, Prummer M, Delobel F, Graff A, Weber M, et al. Disease modeling and phenotypic drug screening for diabetic cardiomyopathy using human induced pluripotent stem cells. Cell Rep. 2014;9:810–20. 10.1016/j.celrep.2014.09.055. 25437537

[13] Carter RD, Purnama U, Castro-Guarda M, Montes-Aparicio CN, Chandran A, Mbasu R, et al. Systems biology and functional assessments of human iPSC-cardiomyocyte models of insulin resistance capture key hallmarks of diabetic cardiomyopathy. Diabetes. 2025;74:1929–45. 10.2337/db25-0204. PMC12585165 40956683

[14] Wang D, Yin Y, Wang S, Zhao T, Gong F, Zhao Y, et al. FGF1∆HBS prevents diabetic cardiomyopathy by maintaining mitochondrial homeostasis and reducing oxidative stress via AMPK/Nur77 suppression. Sig Transduct Target Ther. 2021;6:133. 10.1038/s41392-021-00542-2. PMC7991671 33762571

[15] Suh JM, Jonker JW, Ahmadian M, Goetz R, Lackey D, Osborn O, et al. Endocrinization of FGF1 produces a neomorphic and potent insulin sensitizer. Nature. 2014;513:436–9. 10.1038/nature13540. PMC4184286 25043058

[16] Yang X, Artibani M, Jin Y, Aggarwal A, Zhang Y, Muñoz-Galvan S, et al. 3D Microtumors representing ovarian cancer minimal residual disease respond to the fatty acid oxidation inhibitor perhexiline. Adv Healthc Mater. 2025;14:2404072. 10.1002/adhm.202404072. PMC12118330 39924751

[17] Li N, Zhang C, Xu M, Choi Y, Sidarta-Oliveira D, Dong R et al. Human-derived cardiac-neural microtissues reveal catecholaminergic polymorphic ventricular tachycardia is also a disease of the sympathetic neuron. J Physiol [cited 2026 Feb 16];n/a. 10.1113/JP290024 41691612

[18] Hunter B, Zhang Y, Harney D, McEwen H, Koay YC, Pan M, et al. Left ventricular myocardial molecular profile of human diabetic ischaemic cardiomyopathy. EMBO Mol Med. 2025;17:2483–524. 10.1038/s44321-025-00281-9. PMC12423312 40759793

[19] Upadhyay RK. Emerging risk biomarkers in cardiovascular diseases and disorders. J Lipids. 2015;2015:971453. 10.1155/2015/971453. PMC4407625 25949827

[20] Christoffersen C. Apolipoprotein M—A Marker or an active player in type II diabetes? Front Endocrinol. 2021 [cited 2026 Mar 18];12. 10.3389/fendo.2021.665393 PMC8176018 34093440

[21] Peng M, Fu Y, Wu C, Zhang Y, Ren H, Zhou S. Signaling pathways related to oxidative stress in diabetic cardiomyopathy. Front Endocrinol. 2022 [cited 2026 Mar 18];13. 10.3389/fendo.2022.907757 PMC9240190 35784531

[22] Shirakawa K, Sano M. Osteopontin in cardiovascular diseases. Biomolecules. 2021 [cited 2026 Mar 18];11. 10.3390/biom11071047 PMC8301767 34356671

[23] Zhao Y, Huang Z, Gao L, Ma H, Chang R. Osteopontin/SPP1: a potential mediator between immune cells and vascular calcification. Front Immunol [Internet]. 2024. 10.3389/fimmu.2024.1395596. [cited 2026 Mar 18];15. PMC11196619 38919629

[24] Gordin D, Forsblom C, Panduru NM, Thomas MC, Bjerre M, Soro-Paavonen A, et al. Osteopontin is a strong predictor of incipient diabetic nephropathy, cardiovascular disease, and all-cause mortality in patients with type 1 diabetes. Diabetes Care. 2014;37:2593–600. 10.2337/dc14-0065. 24969575

[25] Hu Y, Zhang Y, Liu Y, Gao Y, San T, Li X, et al. Advances in application of single-cell RNA sequencing in cardiovascular research. Front Cardiovasc Med. 2022;9:905151. 10.3389/fcvm.2022.905151. PMC9360414 35958408

[26] Giardinelli S, Meliota G, Mentino D, D’Amato G, Faienza MF. Molecular basis of cardiomyopathies in type 2 diabetes. Int J Mol Sci. 2024 [cited 2026 Mar 18];25. 10.3390/ijms25158280 PMC11313011 39125850

[27] Zhang D, Xiao W, Liu C, Wang Z, Liu Y, Yu Y, et al. Exosomes derived from adipose stem cells enhance bone fracture healing via the activation of the Wnt3a/β-catenin signaling pathway in rats with type 2 diabetes Mellitus. Int J Mol Sci. 2023 [cited 2026 Mar 18];24. 10.3390/ijms24054852 PMC10003369 36902283

[28] Huang Y, Cai L, Liu X, Wu Y, Xiang Q, Yu R. Exploring biomarkers and transcriptional factors in type 2 diabetes by comprehensive bioinformatics analysis on RNA-Seq and scRNA-Seq data. Ann Trans Med. 2022;10:1017–1017. 10.21037/atm-22-4303. PMC9577746 36267740

[29] Purnama U, Castro-Guarda M, Sahoo OS, Carr CA. Modelling diabetic cardiomyopathy: using human stem cell-derived cardiomyocytes to complement animal models. Metabolites. 2022 [cited 2026 Mar 18];12. 10.3390/metabo12090832 PMC9503602 36144236

[30] Granéli C, Hicks R, Brolén G, Synnergren J, Sartipy P. Diabetic cardiomyopathy modelling using induced pluripotent stem cell derived cardiomyocytes: recent advances and emerging models. Stem Cell Rev Rep. 2019;15:13–22. 10.1007/s12015-018-9858-1. PMC6513824 30343468

[31] Gu W, Li H, Yuan W, Fu X, Wang R, Xu X, et al. Dysfunction of PDE4DIP contributes to LVNC development by regulating cell polarity, skeleton, and energy metabolism via Rho-ROCK pathway. Genes Dis. 2025;12:101568. 10.1016/j.gendis.2025.101568. PMC12221769 40612665

[32] Cribbs JT, Strack S. Reversible phosphorylation of Drp1 by cyclic AMP-dependent protein kinase and calcineurin regulates mitochondrial fission and cell death. EMBO Rep. 2007;8:939–44. 10.1038/sj.embor.7401062. PMC2002551 17721437

[33] Wu Q-R, Zheng D-L, Liu P-M, Yang H, Li L-A, Kuang S-J, et al. High glucose induces Drp1-mediated mitochondrial fission via the Orai1 calcium channel to participate in diabetic cardiomyocyte hypertrophy. Cell Death Dis. 2021;12:216. 10.1038/s41419-021-03502-4. PMC7910592 33637715

[34] Mourmoura E, Vial G, Laillet B, Rigaudière J-P, Hininger-Favier I, Dubouchaud H, et al. Preserved endothelium-dependent dilatation of the coronary microvasculature at the early phase of diabetes mellitus despite the increased oxidative stress and depressed cardiac mechanical function ex vivo. Cardiovasc Diabetol. 2013;12:49. 10.1186/1475-2840-12-49. PMC3620680 23530768

[35] Herring N, Kalla M, Paterson DJ. The autonomic nervous system and cardiac arrhythmias: current concepts and emerging therapies. Nat Rev Cardiol. 2019;16:707–26. 10.1038/s41569-019-0221-2. 31197232

[36] Mattiazzi A, Kranias EG. CaMKII regulation of phospholamban and SR Ca2 + load. Heart Rhythm. 2011;8:784–7. 10.1016/j.hrthm.2010.11.035. PMC3081991 21111063

[37] Kim O-H, Noh SW, Choi J-S, Jung Y, Oh B-C. The SERCA–PLN–DWORF axis in cardiometabolic disease: mechanisms and therapeutic perspectives. Cardiovasc Diabetol. 2025;25:17. 10.1186/s12933-025-03023-w. PMC12814605 41390717

[38] Casis O, Echevarria E. Diabetic cardiomyopathy: electromechanical cellular alterations. Curr Vasc Pharmacol. 2004;2:237–48. 10.2174/1570161043385655. 15320822

[39] Diabetic cardiomyopathy. Where are we 40 years later? Can J Cardiol. 2006;22:305–8. 10.1016/S0828-282X(06)70914-X. PMC2560522 16568154

[40] Jia G, Hill MA, Sowers JR. Diabetic cardiomyopathy. Circul Res. 2018;122:624–38. 10.1161/CIRCRESAHA.117.311586. PMC5819359 29449364

[41] Heather LC, Gopal K, Srnic N, Ussher JR. Redefining diabetic cardiomyopathy: perturbations in substrate metabolism at the heart of its pathology. Diabetes. 2024;73:659–70. 10.2337/dbi23-0019. PMC11043056 38387045

[42] Baillie GS, MacKenzie SJ, McPhee I, Houslay MD. Sub-family selective actions in the ability of Erk2 MAP kinase to phosphorylate and regulate the activity of PDE4 cyclic AMP-specific phosphodiesterases. Br J Pharmacol. 2000;131:811–9. 10.1038/sj.bjp.0703636. PMC1572393 11030732

[43] Li N, Edel M, Liu K, Denning C, Betts J, Neely OC, et al. Human induced pluripotent stem cell-derived cardiac myocytes and sympathetic neurons in disease modelling. Philos Trans R Soc Lond B Biol Sci. 2023;378:20220173. 10.1098/rstb.2022.0173 PMC10150199 37122212

