# Supplementary material for: Compartment-specific cAMP dysfunction in diabetic cardiomyopathy in hiPSC-derived cardiomyocytes: therapeutic potential of fibroblast growth factor 1
Source: Cardiovasc Diabetol. 2026 Jul 30;25:313. doi: 10.1186/s12933-026-03288-9 (PMC13712054; doi:10.1186/s12933-026-03288-9)
Supplement: Supplementary file 1 [file 12933_2026_3288_MOESM1_ESM.docx]

**Supporting information for**

**Compartment-Specific cAMP Dysfunction in Diabetic Cardiomyopathy in hiPSC-derived Cardiomyocytes: Therapeutic Potential of Fibroblast Growth Factor 1**

Kun Liu^1†*^, Kazuki Osuka^1†^, Mengying Xu^2^, Rhea Takhar^1^, Neda Mohammadi^1^, Adam Trevett^1^, Jinyan Zhong^3^, Ching-Wen Chang^4^, Nan Wang^3^, Xinyu Hu^2^, Ruirui Dong^2^, Shunan Zhang^5^, YoonYoung Choi^1^, Thamali Ayagama^1^, Mingyu Li^6^, Linna Zhou^1,6^, Che-Mai Chang^4^, Wan-Chen Huang^7^, Wei-Chiao Chang^4^, Neil Herring^1^, Annika Winbo^8^, Guoliang Hao^2*^, Jie Zhang^3*^, Dan Li^1,9*^

1. Burdon Sanderson Cardiac Science Centre and BHF Centre of Research Excellence, Department of Physiology, Anatomy and Genetics, University of Oxford, Oxford, UK.
2. Institute of Electrophysiology, Henan Academy of Innovations in Medical Science, Zhengzhou 450000, China.
3. Ningbo No.2 Hospital, Wenzhou Medical University. No.41, Xibei Street, Ningbo, Zhejiang, China.
4. Master Program in Clinical Genomics and Proteomics, School of Pharmacy, Taipei Medical University, Taipei, Taiwan.
5. Institute of Medicinal Plant Development, Chinese Academy of Medical Sciences & Peking Union Medical College, Beijing 100193, China
6. Ellison Institute of Technology, Winchester House, Heatley Road, The Oxford Science Park, Littlemore, Oxford, UK.
7. Single-Molecule Biology Core Lab, Institute of Cellular and Organismic Biology (ICOB), Academia Sinica, 128 Academia Road, Section 2, Nankang, Taipei, 115, Taiwan.
8. Department of Public Health and Clinical Medicine, Faculty of Medicine, Umeå University, Umeå, Sweden
9. CAMS Oxford Institute, Chinese Academy of Medical Sciences & Peking Union Medical College, University of Oxford, Oxford, UK.

† Kun Liu and Kazuki Osuka contributed equally to this work.

* Correspondence authors:

Dan Li, [dan.li@dpag.ox.ac.uk](mailto:dan.li@dpag.ox.ac.uk)

Zhang Jie, [hongdou0322zj@163.com](mailto:hongdou0322zj@163.com)

Guoliang Hao, [guoliang.hao@epscopelab.com](mailto:guoliang.hao@epscopelab.com)

Kun Liu, [kun.liu@dpag.ox.ac.uk](mailto:kun.liu@dpag.ox.ac.uk)

**Results**

**Figure S1. Microelectrode array (MEA) analysis of 2D hiPSC-derived cardiomyocytes (CM).** **(A)** Representative field potential traces from control (n=10), DCM (n=6), and DCM+FGF1 (n=9) groups. (**B–E**) Quantification of RR interval, field potential duration (FPD), T-peak to T-end interval (Tpe), and QRS interval (QRSi) at baseline and following 100 nM isoproterenol (ISO) stimulation at 1- and 5-min. FPD, Tpe, and QRSi were analysed only when the corresponding waveform landmarks could be reliably identified; in some recordings, these features were not clearly resolvable. Statistical analysis may be underpowered for panels C-E. Data are presented as mean ± SEM. *P < 0.05; one-way ANOVA; n represents the total number of MEA wells from 3 independent differentiation batches.

**Figure S2.** **Microelectrode array (MEA) anaysis of 2D hiPSC-derived cardiomyocytes (CM) following FGF1 treatment (48 hours). (A)** Representative field potential traces at baseline and following isoproterenol (ISO; 1 and 5 min) in control (n=10) and FGF1-treated (n=5) hiPSC-CMs. **(B–G)** Quantification of spontaneous beat rate, RR interval, amplitude, FPD, Tpe, and QRSi at baseline and following 100 nM ISO stimulation at 1- and 5-min. Note: Technical limitations in measuring T-wave and QRS parameters resulted in reduced sample sizes for panels E-G, limiting statistical power. Data are presented as mean ± SEM. *P < 0.05; one-way ANOVA; n represents the total number of MEA wells from 2 independent differentiation batches.

**Figure S3. Optical mapping of calcium dynamics in 3D hiPSC-derived cardiomyocytes (CM) following FGF1 treatment (48 hours) upon 1Hz stimulation.** Spatial maps of activation **(A)** and calcium transient traces **(B)** from control (n=15) and FGF1-treated (n=15) cardiac microtissues at baseline and following isoproterenol (ISO) stimulation (1 to 7 min) under 1Hz stimulation. (**C-G**) Quantification of active time, amplitude, velocity, rise time, and calcium transient duration at 50% decay (CTD50) from control and FGF1-treated 3D hiPSC-CMs at baseline and following 100 nM ISO stimulation. Data are presented as mean ± SEM. *P < 0.05, **P < 0.01, ***P < 0.001, ****P < 0.0001. one-way ANOVA; n represents the total number of 3D cardiac microtissues from 3 independent differentiation batches.

.

**Figure S4. Optical mapping of calcium dynamics in 3D cardiac microtissues derived from healthy WT hiPSC line at a later maturation stage (day 44).** Quantification of active time **(A),** calcium transient duration at 50% decay (CTD50) **(B)**, transient amplitude **(C),** beat rate **(D),** calcium propagation velocity **(E),** and rise time **(F)** from in control (n=5), DCM (n=6), and DCM+FGF1 (n=5) cardiac microtissues at basal and following 100nM isoproterenol (ISO) stimulation. In this supportive validation dataset, control microtissues showed a clearer isoproterenol-induced increase in beat rate and calcium transient amplitude compared with earlier day 33–35 constructs from Gibco hiPSC line (Figure 10), consistent with maturation-dependent β-adrenergic responsiveness. Data are from a single differentiation batch and should be interpreted as preliminary. Data are mean ± SEM; *P < 0.05, **P < 0.01, ***P < 0.001; one-way ANOVA; n represents the total number of 3D microtissues.

**Figure S5. KEGG pathway diagrams annotated with differential gene expression in DCM relative to control samples.** Gene nodes are coloured according to log₂ fold change, with colour intensity reflecting the magnitude and statistical significance of differential expression. Pathway maps are derived from KEGG and overlaid with RNA-seq results to visualise the spatial distribution of transcriptional changes within each signalling network. **(A) Diabetic cardiomyopathy** and **(B) insulin resistance pathways** exhibit overall activation, consistent with enrichment of metabolic, inflammatory, and stress-response processes. In contrast, **(C) cAMP** and **(D) calcium signalling pathways** show coordinated suppression of key signalling components, particularly those involved in cyclic nucleotide signalling and excitation–contraction coupling.

**Figure S6. Western blot detection of PDE4DIP protein expression in hiPSC-CMs.** Representative western blots probed with anti-PDE4DIP (150 kDa; 1:1000, ab121275, Abcam) and anti-GAPDH (36 kDa; 1:50,000, Proteintech) antibodies. Red arrows indicate target bands. GAPDH serves as the loading control.
